# Greenland sharks conform to expectations of neutral buoyancy but exhibit inter-individual variation

**DOI:** 10.1242/jeb.252246

**Published:** 2026-09-25

**Authors:** Eric Ste-Marie, Yuuki Y. Watanabe, Marianne Marcoux, Nigel E. Hussey

**Affiliations:** ^1^ Department of Integrative Biology, University of Windsor, Windsor, ON, Canada N9B 3P4; ^2^ Research Center for Integrative Evolutionary Science, The Graduate University for Advanced Studies, SOKENDAI, Hayama, Kanagawa 240-0193, Japan; ^3^ Freshwater Institute, Fisheries and Oceans Canada, 501 University Crescent, Winnipeg, MB, Canada R3T 2N6

**Keywords:** Biologging, Arctic, Buoyancy, Behaviour, Sharks, Accelerometers

## Abstract

Buoyancy is an omnipresent force that governs how aquatic animals move through three-dimensional space, leading to physiological and behavioural adaptions across species. Swimming effort during vertical movements is increasingly being used to infer buoyancy in animals that exhibit oscillatory diving behaviours and for which collecting direct measurements of buoyancy is infeasible. Here, we estimated the relative buoyancy of 29 Greenland sharks (*Somniosus microcephalus*) tagged with biologgers by comparing swimming effort [overall dynamic body acceleration (ODBA)/speed] during ascending and descending dives, and accounting for dive geometry. We found that buoyancy in Greenland sharks varied on both sides of the spectrum, with effort in the counter-buoyancy direction reaching as high as 225% of the effort exerted in the buoyancy-aided direction in negatively buoyant individuals, and up to 177% for positively buoyant individuals. Despite this, there was no overall bias towards positive or negative buoyancy for this species, supporting the expectation that slow-moving deep-sea species face selective pressure towards neutral buoyancy. Given that gliding behaviour was rare and that small deviations from neutral buoyancy could drive large differences in effort for a minimally active species, more research is needed to determine the exact magnitude of the buoyancy variation observed here. Even so, its impact on swimming efficiency probably regulates movement and foraging strategies, as sharks are forced to either cope with, or exploit, their tendency to sink or float. These findings also highlight the need for studies directly linking biologger-inferred buoyancy to true buoyancy, with implications for the study of body condition in sharks.

## Teaser

**Summary:** Greenland sharks span both sides of the buoyancy spectrum, but center around neutral buoyancy in accordance with expectations for such a slow-moving species inhabiting cold Arctic waters.

## INTRODUCTION

All animals experience gravity and given they do not exist in a vacuum, must also cope with the effects of buoyancy; a force exerted on an object by a surrounding fluid (e.g. water and air). An object or animal sinks if it is denser than the surrounding fluid and floats if it is less dense. This omnipresent force has far-reaching implications for the behaviour, body shape and physiology of animals, leading to important evolutionary adaptations and trade-offs (Alexander, 1990; Gleiss et al., 2017; Pelster, 2009; Sato et al., 2013; Williams et al., 2000). Terrestrial animals are largely constrained to two-dimensional space because the air which envelops them is much less dense than their bodies, thus giving them weight (Denny, 1990). Water is roughly three orders of magnitude denser than air, allowing many aquatic species to achieve near-neutral buoyancy and exploit three-dimensional space (Pelster, 2009), without the need for costly behavioural adaptations such as flight (Elliott et al., 2013). In fact, the evolution of buoyancy control in early fish was a key factor in their eventual global expansion (Gleiss et al., 2017). Today, fish rely on several strategies to regulate buoyancy, typically involving either gas-filled cavities such as swim bladders, or the accumulation of low-density lipids (Pelster, 2009).

Sharks do not possess swim bladders but instead regulate buoyancy hydrostatically through low-density lipid stores in their livers, or they compensate for it hydrodynamically through lift generated while swimming (Alexander, 1965; Ferry and Lauder, 1996; Iosilevskii and Papastamatiou, 2016). Buoyancy in sharks varies by species, body condition and according to several ecological factors such as habitat type, salinity, depth, and temperature (Gleiss et al., 2015, 2017; Priede et al., 2020; Treberg and Speers-Roesch, 2016). Fast-swimming pelagic sharks have been shown to be predominantly negatively buoyant, using lift generated by their pectoral fins, caudal fins and body to maintain their depth while swimming (Iosilevskii and Papastamatiou, 2016). On the other end of the spectrum, slow-moving, deep or cold-water species have larger fatty livers and are predicted to be closer to neutral buoyancy. The behavioural, energetic and physiological implications of differing buoyancies in sharks is a subject of growing interest, as technological advancements facilitate the study of these elusive fish (Nakamura et al., 2015; Watanabe and Papastamatiou, 2023).

Recently, biologging has become a useful tool to estimate the relative buoyancy of several marine taxa under natural conditions through quantifying swimming effort during ascending and descending vertical dive phases (Gleiss et al., 2019; Nowacek et al., 2001; Sato et al., 2002; Watanabe et al., 2006; Williams et al., 2000). This has allowed researchers to assess buoyancy in the field without the need for captivity or lethal sampling (Gleiss et al., 2011; Nakamura et al., 2011, 2015). For example, depth and speed data from satellite-tagged southern elephant seal pups (*Mirounga leonina*) were used to estimate drift rates – a proxy for buoyancy – which were in turn linked to body condition as individuals depleted lipid stores after weaning (Biuw et al., 2003). In addition, biologged acceleration, depth and swim speed provided novel evidence of positive buoyancy in two species of deep-sea shark [the bluntnose sixgill shark (*Hexanchus griseus*) and the prickly shark (*Echinorhinus cookie*); Nakamura et al., 2015]. The authors calculated swimming effort during ascending and descending dive phases, and showed that these two species consistently exhibited greater swimming effort during descents, countering their tendency to float. Accelerometers have also been applied towards identifying gliding behaviour, whereby animals exploit their buoyancy to move vertically without stroking, thus offsetting some of the high energetic costs of movement in the buoyancy-hindered direction (Miller et al., 2012).

With a maximum recorded body size exceeding 6 m and a geographic range extending from the North Atlantic to the Arctic, the Greenland shark (*Somniosus microcephalus*) is the largest polar shark on the planet (MacNeil et al., 2012). It is a generalist consumer, feeding on a wide variety of prey including invertebrates, fish and marine mammals (Leclerc et al., 2012; McMeans et al., 2013; Nielsen et al., 2019). Yet, inconsistent access to patchily distributed and highly seasonal resources in the Arctic could lead to extreme spatial and temporal variability in body condition (Furey et al., 2016; Lindmark et al., 2023; Ste-Marie et al., 2022), and in turn, buoyancy across Greenland sharks. Although direct assessments of body condition have yet to be undertaken for the species, reported liver mass suggests variation across individuals, with the most notable variation occurring in reproductively mature females (Nielsen et al., 2020). Greenland sharks have been recorded swimming at an average speed of 0.34 m s^−1^, which is among the slowest recorded for any fish relative to body size (Watanabe et al., 2012), and estimation of resting and field metabolic rates demonstrate that this species requires very little energy to survive in their cold Arctic habitats (Ste-Marie et al., 2020, 2022). With such variable body conditions, low overall activity levels and low metabolic demands, buoyancy could disproportionately impact locomotory energetic costs in Greenland sharks when compared with other more active shark species, but the exact degree of variation and its corresponding impact on buoyancy in this species is currently unknown.

The goal of the present study was to use biologged acceleration and depth to infer relative buoyancy across Greenland sharks and assess whether any observed differences were related to size, sex, tagging location or a combination of factors. As a large and slow-moving species typically inhabiting deep sea areas, Greenland sharks are expected to be near neutrally buoyant, but variation in body condition tied to the environment they inhabit could lead to inter-individual differences in buoyancy, ultimately affecting their energetic needs, behaviour and ecology.

## MATERIALS AND METHODS

### Data collection

Greenland sharks [*Somniosus microcephalus* (Bloch & J. G. Schneider 1801)] were captured between 2015 and 2021 with baited circle hooks attached to longlines set for 2–12 h in three locations across the Canadian and Norwegian Arctic: Scott Inlet, Nunavut (70.88, –71.49), Tremblay Sound, Nunavut (72.34, −81.12) and Kongsfjorden, Svalbard (79.00, 11.46). Once captured, animals were secured next to a small research vessel using straps around the body and measured [total length (TL) and fork length (FL)], tissue sampled (fin clips, blood and eye parasites) and subsequently equipped with electronic tags for separate tracking studies (Edwards et al., 2022). A subset of sharks were also equipped with biologger packages recording depth, temperature and triaxial acceleration [DTAG-3 (Johnson and Tyack, 2003), Sonar tag (Goulet et al., 2019), PD3GT Little Leonardo; MBLog tri-axial accelerometer, Maritime BioLoggers] using a cable tie release system (Little Leonardo) inserted underneath the skin between the head and the dorsal fin. Biologgers were deployed for durations ranging from less than 24 h to approximately 6 days, after which the cable tie was programmed to release the tag package from the animal (Watanabe et al., 2004), allowing it to float to the surface and be recovered using signals from VHF radio transmitters (Advanced Telemetry Systems) and SPOT tags (Wildlife Computers) that were integrated into the tag packages. See Ste-Marie et al. (2022) for a more detailed description.

All fieldwork was done with approval from Canadian and Norwegian authorities, as well as from the University of Windsor's Animal Care Committee (AUPP #18-01, #17-12 and #25-05).

### Biologged data processing

All biologged data were initially processed in Igor Pro (Wavemetrics, Lake Oswego, OR, USA) using the Ethographer extension (Sakamoto et al., 2009). Dynamic body acceleration in the heave, sway and surge axes was derived from raw triaxial acceleration by subtracting the static component of acceleration in each axis (i.e. the acceleration resulting from gravity and body position). Static acceleration for each axis was obtained by applying a rolling mean to the raw acceleration data. Smoothing windows should be at least one complete caudal fin stroke cycle in duration in order to remove the acceleration signal originating from the animal's swimming (Shepard et al., 2008). As a very slow swimming species with low tail beat frequencies (Watanabe et al., 2012), 10 s was deemed appropriate for Greenland sharks. Overall dynamic body acceleration (ODBA) was then calculated for each tagged shark by summing the absolute values of the dynamic component of acceleration in all three axes. Depth was smoothed using a 10 s rolling average and then used to derive vertical velocity (*v_y_*); the central difference in smoothed depth calculated at one second intervals (Whitney et al., 2016). The angle (or pitch) of accelerometer tags throughout each deployment was determined by calculating the arcsine of static acceleration in the surge axis (head-to-tail). Shark pitch over time was then determined by subtracting the intercept of a linear relationship between tag pitch and *v_y_* from tag pitch (Kawatsu et al., 2009). Tail beat cycle (TBC) was derived from the dynamic component of acceleration in the sway axis using continuous wavelet transformation. The first 10 h of each deployment was excluded from analysis to account for potential capture effects on swimming behaviour (Watanabe et al., 2026), and the data were resampled to a more biologically relevant rate of 0.1 Hz.

Periods of persistent ascending and descending vertical movement by Greenland sharks were identified using *v_y_* such that a shark was considered to be ascending if *v_y_* was greater than 0.03 m s^−1^ and descending if *v_y_* was less than −0.03 m s^−1^. This threshold was chosen after visual inspection of the depth time series data revealed the standard threshold of 0.05 m s^−1^ used to identify dives in several shark species was insufficient to capture all periods of diving in this uniquely slow-moving species (Andrzejaczek et al., 2019; Gleiss et al., 2011; Whitney et al., 2016). Given that bouts of ascending and descending diving can vary in length and that individual data points within dives are autocorrelated, data points were grouped into dive segments and averaged prior to analysis. Dive segments were defined as sequences of consecutive points covering a depth change of at least 5 m in which a shark remained consistently in either the ascending or descending state. As a result of this relatively conservative threshold, all short or irregular dives were excluded from the buoyancy analysis in addition to periods of level swimming.

### Relative buoyancy

In species exhibiting yo-yo diving behaviour, relative buoyancy can be inferred by comparing the swimming effort of individuals during periods of continuous ascent and descent (Gleiss et al., 2011). Negatively buoyant individuals are expected to increase swimming effort during ascents to counteract the downward force resulting from their weight in water, while positively buoyant individuals increase effort during descents to counteract the experienced upward force. Here, we compare the mean ODBA exhibited by sharks during ascents and descents throughout biologger deployments to infer buoyancy. To ensure differences in ODBA during bouts of diving were related to buoyancy, we standardized ODBA by dividing it by estimated swim speed [i.e. speed=*v_y_*/sin(pitch); Sato et al., 2003]. As such, swimming effort during dives was defined as the ODBA required to move at a speed of 1 m s^−1^. Speed was only estimated for dives with an average pitch of greater than 5 deg for ascents and less than −5 deg for descents, as angles close to zero are more likely to lead to erroneous estimates (Gleiss et al., 2011). This value was a lower cutoff than used in previous studies (e.g. 20 deg in Gleiss et al., 2011), but was based on direct inspection of the estimated mean and maximum speeds for all Greenland sharks to derive a plausible estimate (Fig. S1; Watanabe et al., 2012). Additionally, we: (i) reduced the likelihood of erroneous estimates by calculating the average speeds of entire dive segments instead of on a point-by-point basis where noise in the instantaneous pitch and *v_y_*  could be problematic; and (ii) showed that the coefficient of variation for speed estimates did not increase at lower pitch angles (Fig. S2).

### Statistical analyses

We compared swimming effort (ODBA/speed) during continuous ascents and descents for each individual shark through a series of gamma log link generalized linear models (GLMs) using the stats package in R (r-project.org). Dive geometry has the potential to confound comparisons of swimming effort during dives, given sharks could compensate for buoyant forces by altering the angles of their dives. Consequently, GLMs assessed the effect of the absolute value of pitch on relative swimming effort for each individual shark while accounting for a potential interaction between absolute pitch and the direction of dives (ascending or descending). In other words, the model estimated separate slopes for each dive direction with a common intercept (i.e. swimming effort is the same for ascents and descents when pitch=0). Sharks represented by fewer than 20 ascending dive segments and 20 descending dive segments were excluded from analyses owing to insufficient data relative to the complexity of the model (Bolker et al., 2009). To evaluate the importance of dive direction's effect on the slope, we compared the more complex model above to a simpler formulation without the interaction term using a likelihood ratio test using the anova function in R. If the model including dive direction's effect on the slope was favoured with non-overlapping 95% confidence intervals for the ascending and descending slopes, we assumed those slope differences were indicative of non-neutral buoyancy (i.e. either positive or negative buoyancy depending on which slope was steeper). Sharks not meeting these criteria were labelled as near-neutrally buoyant.

To facilitate comparisons across individuals, we calculated the ratios of ascending to descending swimming effort estimated by the GLMs assuming a fixed pitch of 14.3 deg – the average dive angle exhibited by Greenland sharks across all dives in the dataset. Ratios of greater than 1:1 coincided with negative buoyancy, whereas lower ratios indicated positive buoyancy. We tested whether these directional swimming effort ratios were related to sex, body size or tagging location with basic linear models. To then determine whether the buoyancy of Greenland sharks was biased towards the positive or negative sides of the spectrum at the sample population level, we tested: (i) whether Greenland sharks, on average, deviated significantly from neutral buoyancy using a two-sided Wilcoxon signed rank test comparing the log-transformed effort ratios for all sampled sharks to a value of zero; and (ii) for a linear relationship between mean ascending and mean descending dive angles across all individuals, as non-neutrally buoyant species often exhibit pitch asymmetry across dive directions to minimize transport costs (Andrzejaczek et al., 2020; Gleiss et al., 2011).

Theory suggests sharks with high non-neutral buoyancy are expected to exhibit gliding behaviour in the buoyancy aided dive direction to minimize roundtrip energetic costs (Miller et al., 2012). Here, we estimated the percentage of time when sharks did not use active tail beats during dives, which we labelled as gliding. Since short periods of gliding could result from active tail beats immediately preceding glides and not necessarily from buoyancy, we further isolated individuals who exhibited prolonged periods of gliding (>5 min) and summarized their dive characteristics (i.e. mean±s.d. pitch and *v_y_*).

## RESULTS

In total, biologger datasets for 32 Greenland sharks were considered (Table 1). Three individuals were removed for having small numbers of both ascending and descending dive segments (i.e. fewer than 20 each), leaving 29 individuals for subsequent analysis. The remaining individuals had average sample sizes of 155±101 ascending dive segments and 153±100 descending dive segments. Of these, 26 were tagged in in Nunavut, whereas three individuals were tagged in Svalbard. The sample included both males and females ranging in total length from 163 to 370 cm, including large juveniles and subadults of both sexes, and mature males.

**Table 1.** Summary table of individual Greenland shark (*Somniosus microcephalus*) biologger data sets (*n*=32) with the overall mean for each metric presented in bold at the bottom

Shark ID	Location	Year	Sex	TL (cm)	Ascending dive segments (*n*)	Descending dive segments (*n*)	Depth (m)	Temperature (°C)
GS01_SCOT_2015	Scott Inlet	2015	M	256	32	33	405±49	0.94±0.14
GS02_SCOT_2015	Scott Inlet	2015	F	300	14	13	113±103	0.44±1.55
GS04_SCOT_2015	Scott Inlet	2015	M	222	100	106	388±34	0.92±0.12
GS03_SCOT_2015	Scott Inlet	2015	F	330	77	89	355±87	0.71±0.59
GS05_SCOT_2015	Scott Inlet	2015	M	300	238	224	404±84	0.80±0.43
GS02_SCOT_2016	Scott Inlet	2016	M	312	279	276	428±170	0.82±0.92
GS05_SCOT_2016	Scott Inlet	2016	M	163	133	135	356±129	1.10±0.70
GS04_SCOT_2016	Scott Inlet	2016	M	223	44	44	419±55	1.00±0.19
GS01_TREM_2017	Tremblay Sound	2017	F	292	159	139	87±33	−1.25±0.04
GS09_TREM_2017	Tremblay Sound	2017	M	313	328	318	101±40	−0.87±0.07
GS14_TREM_2017	Tremblay Sound	2017	M	290	262	227	127±50	−1.28±0.05
GS21_TREM_2017	Tremblay Sound	2017	M	286	292	297	68±30	−1.03±0.53
GS22_TREM_2017	Tremblay Sound	2017	M	320	267	236	125±101	−0.49±0.67
GS28_TREM_2017	Tremblay Sound	2017	F	265	111	113	204±135	−0.66±0.85
GS06_TREM_2018	Tremblay Sound	2018	M	276	73	73	65±79	−0.32±0.61
GS07_TREM_2018	Tremblay Sound	2018	M	279	34	71	159±84	−1.45±0.53
GS18_TREM_2018	Tremblay Sound	2018	F	238	146	119	103±53	−0.94±0.13
GS19_TREM_2018	Tremblay Sound	2018	M	288	194	180	369±125	0.44±0.70
GS22_TREM_2018	Tremblay Sound	2018	M	257	140	153	155±89	−0.62±0.59
GS12_TREM_2019	Tremblay Sound	2019	F	322	106	98	63±51	−0.44±0.70
GS11_TREM_2019	Tremblay Sound	2019	M	305	75	84	64±42	−0.42±0.69
GS14_TREM_2019	Tremblay Sound	2019	M	315	87	81	54±60	0.06±0.96
GS15_TREM_2019	Tremblay Sound	2019	F	305	58	44	84±68	−0.10±1.03
GS16_TREM_2019	Tremblay Sound	2019	M	270	76	69	69±45	−0.59±0.65
GS18_TREM_2019	Tremblay Sound	2019	M	285	14	14	70±9	−1.15±0.03
GS19_TREM_2019	Tremblay Sound	2019	F	248	96	80	91±36	−0.90±0.38
GS20_TREM_2019	Tremblay Sound	2019	M	281	54	55	37±33	0.80±1.27
GS21_TREM_2019	Tremblay Sound	2019	M	297	99	98	60±42	−0.29±1.00
GS23_TREM_2019	Tremblay Sound	2019	M	324	9	9	51±46	3.16±3.58
GS01_SVAL_2021	Svalbard	2021	F	297	256	269	141±42	3.20±0.26
GS02_SVAL_2021	Svalbard	2021	F	370	425	439	148±90	3.48±0.75
GS05_SVAL_2021	Svalbard	2021	F	290	262	273	137±73	3.48±0.79
**Mean±s.d.**				**285±38**	**142±105**	**139±104**	**172±132**	**0.27±1**

Results of individual GLMs testing for the effect of absolute pitch on relative swimming effort were significant for 28 of the 29 sharks (Table 2, Fig. 1). Likelihood ratio tests also demonstrated support for the inclusion of the interaction term with dive direction when compared with simplified models without the interaction term in 27 of the 29 sharks. Among these individuals, there were 22 cases in which slope estimates for ascending and descending dives had non-overlapping 95% confidence intervals; 14 with higher ascending slopes (i.e. negative buoyancy) and 8 with higher descending slopes (i.e. positive buoyancy). Individual ratios of ascending to descending swimming effort predicted by GLMs at a set pitch of 14.3 deg ranged from 0.566 to 2.25 (mean±s.d.=1.15±0.410; Fig. 2). Sex (*P*=0.942), tagging location (*P*=0.2122), total length (*P*=0.3525) or a combination of the three (*P*=0.4084) did not significantly explain ratios of ascending to descending swimming effort. Although there was notable variation among individuals, a Wilcoxon signed rank test found that log-transformed effort ratios across Greenland sharks were not significantly different from 0 at the sample population level (*n*=29, *P*=0.3927). The relationship between average ascending and descending pitch across individuals was significant with a slope of near 1 (slope=1.015, *R*^2^=0.6803, *P*=3.734e–08; Fig. 3).

**Fig. 1.**
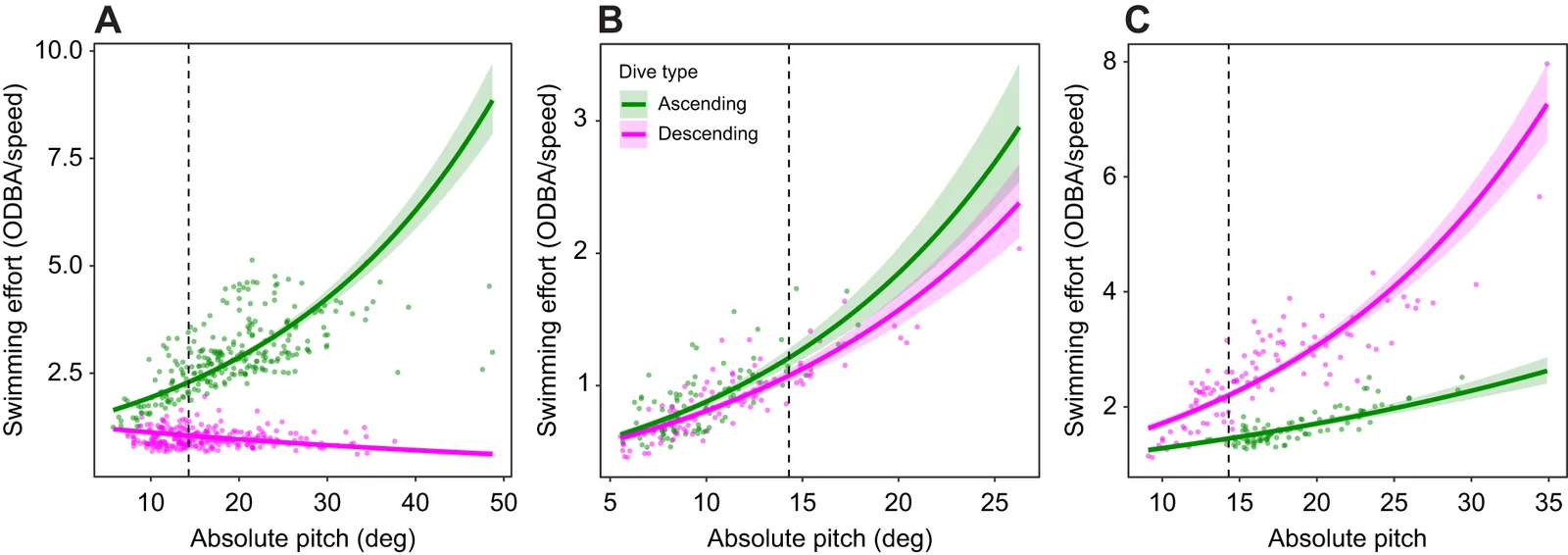
**Swimming effort of Greenland sharks (*Somniosus microcephalus*) during ascents and descents of varying absolute pitch.** Trendlines and corresponding shaded regions represent the output of individual GLMs with 95% confidence intervals. The dashed vertical line indicates an absolute pitch of 14.3 deg (i.e. value used to estimate relative buoyancy ratios for comparisons across individuals based on the average across all sharks). (A) An example of a shark exhibiting swimming behaviour consistent with negative buoyancy (GS05_SVAL_2021). (B) An example of a shark exhibiting signs of neutral buoyancy (GS12_TREM_2019). (C) An example of a shark with swimming behaviour consistent with positive buoyancy (GS04_SCOT_2015). Plots for all other individuals are available in Fig. S3. Note that axes are presented with individual shark-specific scales.

**Fig. 2.**
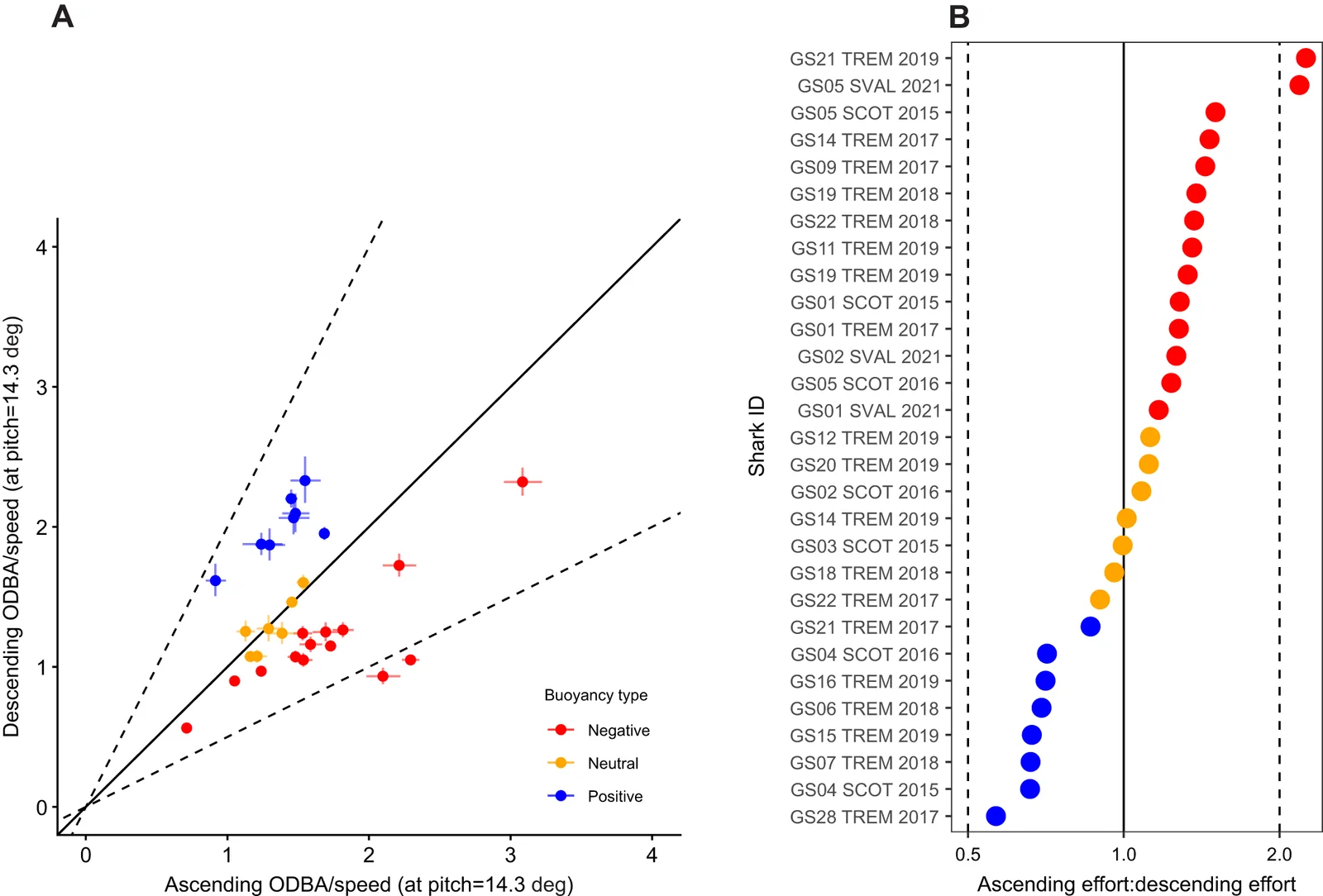
**Pitch-adjusted directional swimming effort and relative effort ratios for 29 Greenland sharks (*Somniosus microcephalus*).** (A) Swimming effort (ODBA/speed) during ascending and descending dive phases predicted by GLMs at an average absolute pitch of 14.3 deg. Point colour represents the assigned relative buoyancy category (i.e. blue, positive; red, negative; yellow, neutral) and whiskers represent 95% confidence intervals. The solid line represents the line of equality, while the two dashed lines indicate when descending effort is twice as high ascending effort (top) and when ascending effort is twice as high as descending effort (bottom). (B) Pitch-adjusted ratios of ascending to descending swimming effort of individual sharks presented in increasing order for visual clarity. The *x*-axis is presented on a log scale so that distances on the right and left of the line of equality represent the same relative difference in swimming effort.

**Fig. 3.**
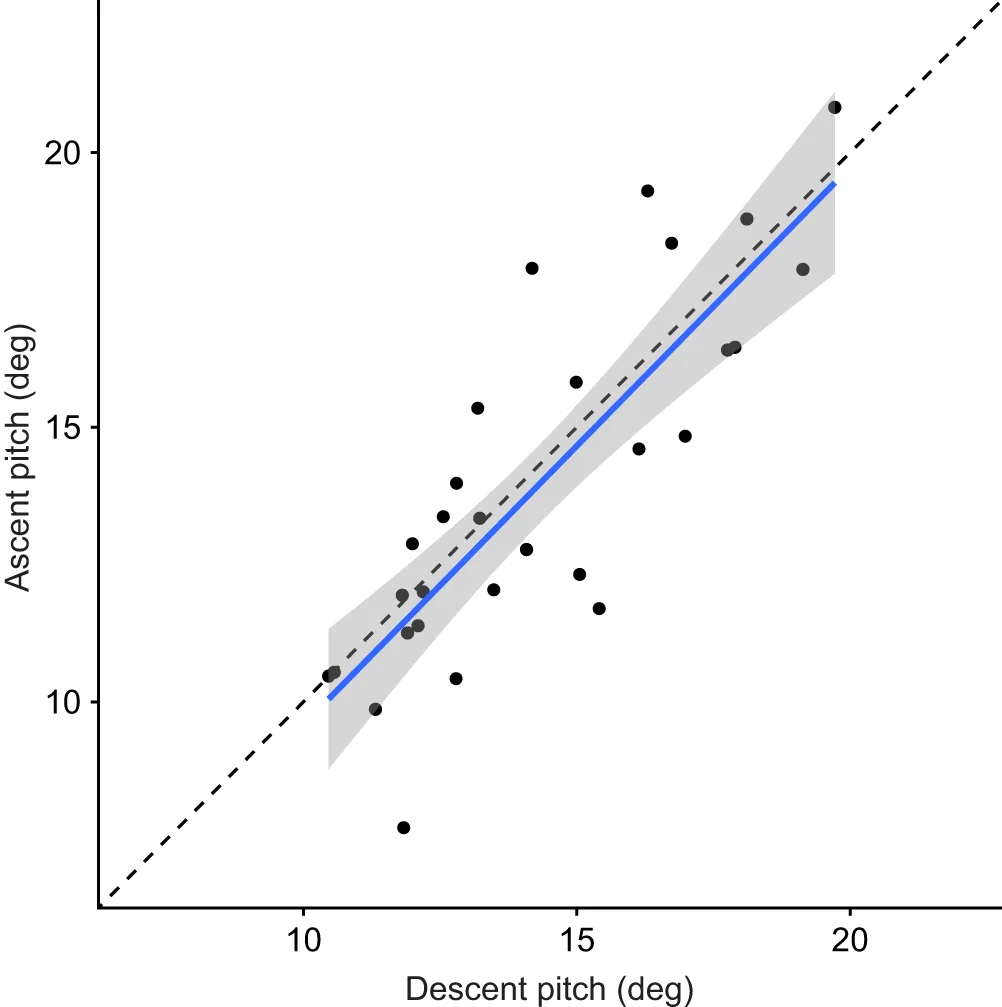
**Scatterplot depicting the mean ascending and descending pitch of individual Greenland sharks (*n*=29).** The solid blue line represents the fit of the linear relationship between both variables (*R*^2^=0.6803, *P*=3.734e–08) and the dashed line depicts the line of equality.

**Table 2.** Summary of individual generalized linear models assessing the effect of pitch on swimming effort in Greenland sharks (*Somniosus microcephalus*; *n*=29) across dive directions (i.e. ascending and descending)

Shark ID	Intercept	Absolute pitch slope	Dive direction slope interaction	Likelihood ratio test
GS01_SCOT_2015	0.171322*	0.04356*	−0.01742*	*
GS04_SCOT_2015	−0.04^n.s.^	0.028814*	0.029159*	*
GS03_SCOT_2015	−0.32311*	0.048832*	0.000343^n.s.^	n.s.
GS05_SCOT_2015	−0.03012^n.s.^	0.040375*	−0.02854*	*
GS02_SCOT_2016	−0.52963*	0.047519*	−0.0055*	*
GS05_SCOT_2016	−0.22486*	0.045522*	−0.01479*	*
GS04_SCOT_2016	0.259422*	0.008658^n.s.^	0.023886*	*
GS01_TREM_2017	−0.55548*	0.0538*	−0.01714*	*
GS09_TREM_2017	−0.00116^n.s.^	0.041799*	−0.02535*	*
GS14_TREM_2017	−0.89692*	0.092747*	−0.02668*	*
GS21_TREM_2017	−0.30563*	0.057831*	0.010331*	*
GS22_TREM_2017	−1.21334*	0.093259*	0.00739*	*
GS28_TREM_2017	−0.74379*	0.045824*	0.039759*	*
GS06_TREM_2018	−0.01678^n.s.^	0.019391*	0.02558*	*
GS07_TREM_2018	−0.02567^n.s.^	0.016805*	0.028999*	*
GS18_TREM_2018	−0.13808*	0.039644*	0.002981*	*
GS19_TREM_2018	−0.50562*	0.062794*	−0.02259*	*
GS22_TREM_2018	−0.79309*	0.087757*	−0.02189*	*
GS12_TREM_2019	−0.87536*	0.07453*	−0.00822*	*
GS11_TREM_2019	−0.40664*	0.065284*	−0.02129*	*
GS14_TREM_2019	−0.53177*	0.055005*	−0.00093^n.s.^	n.s.
GS15_TREM_2019	0.101329^n.s.^	0.023477*	0.028603*	*
GS16_TREM_2019	−0.33695*	0.050987*	0.024336*	*
GS19_TREM_2019	0.758351*	0.025737*	−0.01988*	*
GS20_TREM_2019	−0.64985*	0.068262*	−0.0078*	*
GS21_TREM_2019	0.143095*	0.041818*	−0.05667*	*
GS01_SVAL_2021	−0.41983*	0.03282*	−0.01086*	*
GS02_SVAL_2021	−1.25333*	0.063892*	−0.01636*	*
GS05_SVAL_2021	0.268879*	0.039245*	−0.05465*	*

Note that the models assume ascending as the baseline dive direction. Resulting coefficients are presented alongside n.s. (not significant) or * (*P*<0.05), indicating significance. The LRT column indicates whether the results of a likelihood ratio test support the inclusion of the dive direction slope interaction term.

Gliding was not common during periods of diving in the majority of sampled sharks [mean±s.d. (range)=1.34±2.41 (0, 9.66) per cent of time]. Only three sharks exhibited prolonged glides lasting longer than 5 min (Table 3, Fig. 4).

**Fig. 4.**
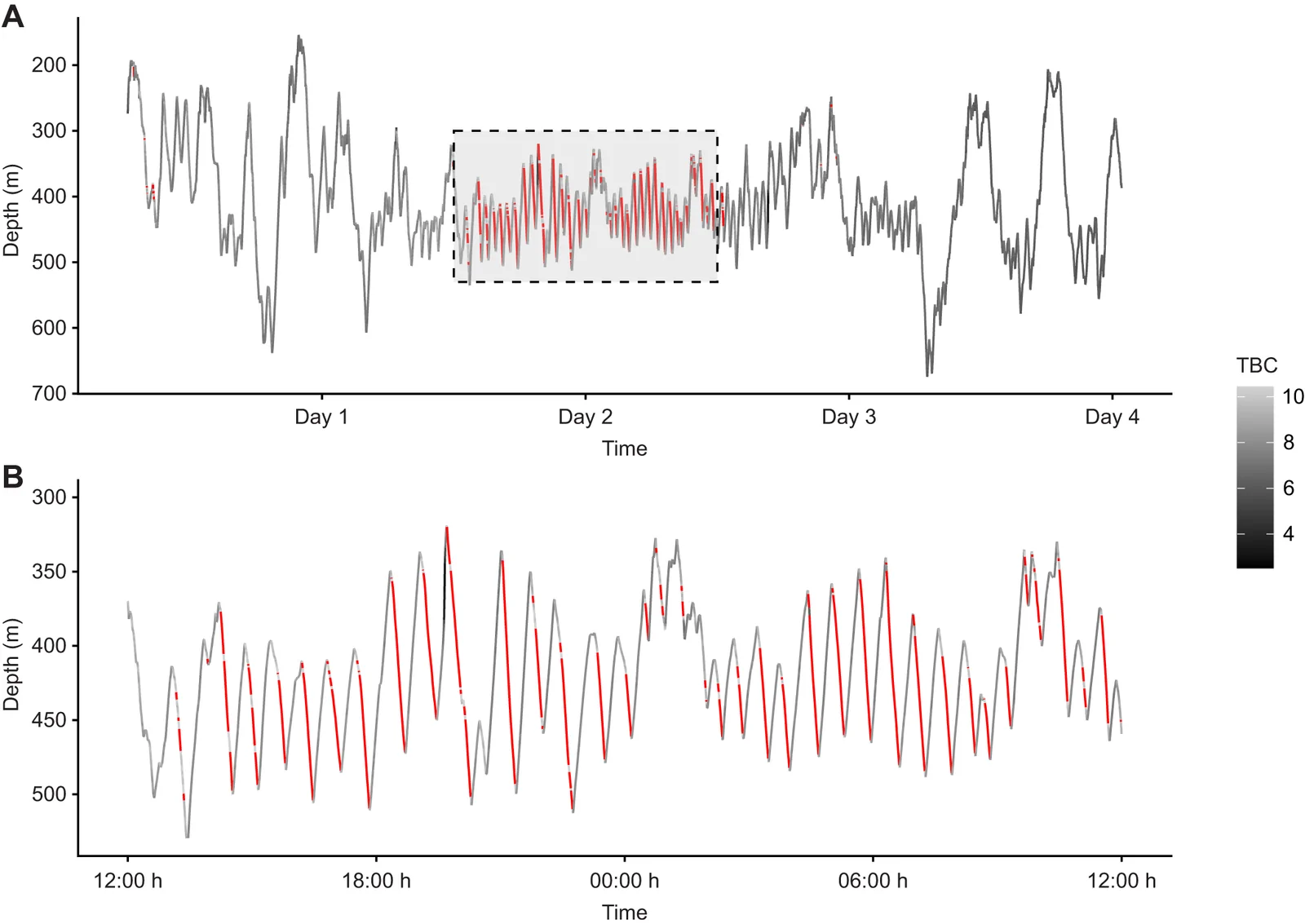
**Time series plots depicting the depth profile of a negatively buoyant Greenland shark (GS05_SCOT_2015) that exhibited gliding behaviour during the descent phases of several dives.** (A) The entire deployment (excluding the 10 h recovery period). Periods with no tailbeats are indicated in red, including some prolonged periods of >5 min. (B) Zoom of the portion of the deployment enclosed by the dashed rectangle in A. For non-gliding periods, line colour varies from black to light grey according to increasing tail beat cycle (TBC).

**Table 3.** Overall vertical velocities and pitch while diving for the three negatively buoyant Greenland sharks that exhibited prolonged periods of gliding (>5 min) in the buoyancy enabled direction

Shark ID	Long glides (>5 min)		Descending dives		Ascending dives	
	*v_y_* (m s^−1^)	Pitch (deg)	*v_y_* (m s^−1^)	Pitch (deg)	*v_y_* (m s^−1^)	Pitch (deg)
GS01_SVAL_2021	−0.13±0.02	−31.3±2.40	−0.07±0.03	−16.3±7.76	0.08±0.04	16.1±7.67
GS05_SCOT_2015	−0.11±0.01	−28.7±2.44	−0.09±0.03	−19.4±7.50	0.09±0.04	17.7±7.94
GS05_SVAL_2021	−0.08±0.01	−17.1±3.52	−0.08±0.04	−18.5±8.53	0.10±0.06	20.9±9.88

Dive statistics (means±s.d.) are provided for gliding specifically and for descending and ascending dives. *v_y_*, vertical velocity.

## DISCUSSION

Behavioural assessments of buoyancy have largely supported the general pattern of shark species ranging from negative to neutral buoyancy in their natural habitats (Gleiss et al., 2017), with one study finding evidence of positive buoyancy in two deep sea sharks (Nakamura et al., 2015). The analyses presented here broadly support expectations of neutral buoyancy for a large slow-moving shark inhabiting polar waters. Across all sampled Greenland sharks, directional swimming effort ratios were not significantly biased towards either negative or positive buoyancy. Limited evidence of buoyancy-aided gliding in most individuals and a lack of asymmetry in mean ascending and descending pitch angles across individuals further reinforced the idea that Greenland sharks likely evolved to be near-neutrally buoyant. At the individual level, however, Greenland shark effort ratios varied substantially, with swimming effort in the counter-buoyancy direction reaching as high as 225% of the effort exerted in the buoyancy-enabled direction in negatively buoyant individuals and as high as 177% for positively buoyant individuals. Although buoyancy-aided gliding was found to be rare, its occurrence in certain individuals, suggests observed effort differences during dives reflect real buoyancy differences. Body size, sex and tagging location did not explain the observed variation across individuals, but we acknowledge that small sample sizes within subgroups and possible interactions between variables could have masked any effects.

### Energetics of diving

Variable buoyancy leads to variable energetic costs of movement and differing optimal swimming strategies (Gleiss et al., 2015; Iosilevskii et al., 2012; Iosilevskii and Papastamatiou, 2016). The ecological drivers of movement (e.g. foraging, thermoregulation, migration, etc.) pose constraints on direct energy minimization, so sharks must balance their various needs against the effort required to meet them (Iosilevskii et al., 2012; Nathan et al., 2008). For negatively buoyant sharks exhibiting yo-yo diving behaviour, minimally active descents aided by gravity (i.e. gliding) and active ascents at differing optimal pitch angles are generally expected to be the most efficient (Gleiss et al., 2011; Weihs, 1973). The opposite should be true for positively buoyant sharks (Nakamura et al., 2015). However, trade-offs between mechanical efficiency and efficiency in other aspects of their lives can support different optimal swimming strategies or physical adaptations that depend on the relative importance of key physiological and ecological drivers (Iosilevskii et al., 2012; Iosilevskii and Papastamatiou, 2016; Saraiva et al., 2023).

Evidence of gliding in Greenland sharks has previously been found to be rare, representing zero to 12% of descent times for sharks tagged in one study (Watanabe et al., 2012). Similarly, the behaviour was not widespread in our sample of sharks and predominantly observed in individuals deemed negatively buoyant by our analyses. If sharks were optimizing their diving behaviour to minimize their overall cost of transport, then gliding would be expected to occur in all sharks whose buoyancy deviated sufficiently from neutral to offset drag forces in the buoyancy-aided direction (Miller et al., 2012). The overall lack of gliding evidence (i.e. <10% of time spent diving in all sharks) and the near 1:1 relationship between ascending and descending pitch across assessed sharks could therefore indicate that deviations away from neutral buoyancy in our sample were relatively small overall (Miller et al., 2012). Other factors, however, can influence the tendency of sharks to exhibit gliding and were likely at play (Saraiva et al., 2023). For example, one of the Greenland sharks with the strongest evidence of prolonged gliding in our sample did not exhibit the behaviour during all descending dives and appeared to restrict its use of gliding to an approximate 24 h window in the middle of its deployment (Fig. 4), suggesting a trade-off between minimizing diving costs and other drivers of activity (e.g. foraging). Interestingly, none of the positively buoyant Greenland sharks showed signs of prolonged gliding in the buoyancy-aided direction, including those with directional swimming effort ratios of similar magnitude (but opposite direction) to the negatively buoyant sharks that did glide. Given the asymmetry between the ventral and dorsal sides of their bodies, it is possible that the shark's morphology enables more biomechanically efficient gliding when the buoyancy-aided direction is down as opposed to up, leading to a higher buoyancy threshold for gliding in positively buoyant sharks. However, we cannot discount the possibility that the observed gliding discrepancy between positively and negatively buoyant sharks was due to chance, especially given the rarity of the behaviour overall.

An important caveat in studies that rely on comparisons of directional effort to infer buoyancy, is that it can be difficult to fully resolve how these assessments translate to true buoyancy (Gleiss et al., 2011; Nakamura et al., 2015; Watanabe et al., 2019). Small differences in actual buoyancy would be expected to lead to relatively large differences in swimming effort and, in turn, estimates of relative buoyancy for slow-moving species such as the Greenland shark (Iosilevskii and Papastamatiou, 2016). As a minimally active ectotherm inhabiting near-freezing waters, counteracting buoyant forces may represent a larger proportion of their overall locomotory effort, possibly contributing to the large effort gaps observed between ascents and descents in some individuals. Ultimately, the exact magnitude of their departure from neutrality would require corroborating body density data, but the occurrence of prolonged gliding in some individuals suggests notable deviations away from neutral buoyancy exist in Greenland sharks.

The ecological implications of the wide spectrum of buoyancy-linked swimming effort observed in Greenland sharks may have complex effects on their feeding behaviour, because different foraging strategies are likely to be more efficient under opposing buoyancy regimes. Buoyancy constraints have already been shown to affect predation dynamics in several marine species through metabolic trade-offs and changes to the movement abilities of individuals. For example, elephant seals have been shown to adjust the duration of their foraging dives in accordance with their body condition, such that neutrally buoyant seals with higher blubber content expend less energy diving and can thus spend more time foraging at depth than more negatively buoyant seals (Adachi et al., 2014). Imperial shags can achieve higher speeds pursuing prey in deeper waters where they are less buoyant and must therefore invest less energy countering their tendency to float (Shepard et al., 2010). In contrast, Magellanic penguins exploit their positive buoyancy to passively pursue pelagic fish during ascending dive phases (Wilson et al., 2010). In Greenland sharks, a tendency to float could aide the pursuit of prey at the surface if sharks are approaching from below, whereas a tendency to sink could give sharks an advantage when pursuing demersal species. Given the varied diet and slow swim speeds of Greenland sharks, as well as the currently unknown strategies with which they capture and consume their prey (Edwards et al., 2019), future studies should examine links between buoyancy and foraging behaviour. Specifically, research should focus on whether and to what extent individuals are able to vary their feeding behaviour and the types of prey they consume in accordance with the constraints or advantages afforded to them by their buoyancy.

### Drivers of buoyancy variation

There are several physiological and environmental factors that could have contributed to the significant inter-individual variation in buoyancy observed in Greenland sharks. The size and lipid composition of livers are largely considered the primary drivers of buoyancy regulation in sharks, with larger low-density livers offsetting higher density tissues such as muscle (Gleiss et al., 2017; Pinte et al., 2019). While neither liver morphology nor composition have been studied extensively in Greenland sharks, one study reported hepatosomatic indices (HSIs) ranging from 7.4% to 20.4% in 42 mostly immature specimens sampled in Svalbard, Norway (Molde et al., 2013). Their livers are also thought to consist primarily of low density triacylglycerols (TAGs) and diacylglyceryl ethers (DAGEs), which, unlike the squalene occurring at high concentrations in many shark species, are metabolically active and potentially subject to greater variability when sharks are faced with spatial or temporal gaps in resource availability (MacNeil et al., 2012; Musick and Cotton, 2015). Other factors such as relative muscle volume, osmolyte concentrations, salinity, depth and temperature have all been shown to affect buoyancy in sharks, but generally to a much lesser extent than changes to the liver (Corner et al., 1969; Gleiss et al., 2015; Pinte et al., 2019; Priede et al., 2020; Treberg and Speers-Roesch, 2016; Withers et al., 1994). Also notable, there were instances where our analyses estimated opposing relative buoyancies (i.e. positive and negative) for similarly sized Greenland sharks tagged in the same location within a couple days of each other and which, therefore, probably experienced similar environmental gradients.

Greenland sharks are known to feed on a variety of species, some of which are marine mammals with thick layers of lipid-rich blubber (Fisk et al., 2002; Leclerc et al., 2012; McMeans et al., 2013; Nielsen et al., 2019). Lipids in blubber, similarly to those in shark livers, are lower in density than seawater and thus could provide a temporary source of positive buoyancy to sharks that consume it. Studies on the diet of Greenland sharks have reported dissected individuals which contained large chunks of blubber in their stomachs (Nielsen et al., 2019), potentially reducing their net body density. Given our lack of knowledge regarding the stomach contents of sharks at the time of tagging, we cannot comment on what impact feeding on blubber may have had on estimated Greenland shark buoyancy. When considering the fragmented distribution of resources in the Arctic and the known voraciousness of Greenland sharks when scavenging on large-bodied mammalian prey (N.E.H. and E.S.-M., pers. obs.), it is feasible that an individual shark could feed on a large mass of blubber in one event leading to modified buoyancy for the period of digestion. Future work would need to ascertain stomach fullness, diet type and digestion speed to verify this, as well as extend the duration of biologger deployments to fully encompass the post meal digestion period. Nevertheless, it is an important factor to consider since it reflects an often-overlooked source of buoyancy variation in large predatory sharks that could confound links between behaviourally inferred buoyancy and liver condition, especially when tag deployments are short.

Variation in the size and composition of livers is widely considered the primary cause of buoyancy differences in sharks, but this is not the only factor influencing buoyancy. One of the potential benefits of using biologgers to assess buoyancy in sharks is that it might provide a non-lethal way to estimate their liver condition, and by proxy, overall body condition; something that is notoriously challenging to do in live sharks (Gallagher et al., 2014; Hussey et al., 2009; Irschick and Hammerschlag, 2014). To achieve this, future research should focus on confirming that biologger-derived buoyancy is indeed correlated to liver condition while accounting for confounding factors such as stomach contents and salinity. The latter consideration is simple to address since salinity loggers are already widely available. To address the former, ultrasonography – a technology which is becoming increasingly available to field biologists – could be used to evaluate shark liver condition and determine stomach contents in animals prior to tagging and release, pending adequate validation of the method (Grant et al., 2013; Hussey et al., 2009; Schilling et al., 2025).

### Conclusion

Natural selection has led to a spectrum of buoyancy regulation strategies in sharks. Whereas fast pelagic species have largely evolved sleek bodies with hydrofoil-like fins to counter their weight in water, slow deep-water species have evolved large lipid-rich livers to maintain neutral buoyancy. Among the slowest fish in the ocean, Greenland sharks represent an example of the latter group, but considerable variation in directional swimming effort during dives across individuals suggest a degree of negative and positive buoyancy occurs within the species. Our findings add to the growing body of literature using biologging to study the buoyancy of sharks and provide the first evidence of a shark species with individuals occupying both sides of the buoyancy spectrum. The broad implications of this on the behaviour and ecology of Greenland sharks, and our ability to assess the health and condition of sharks in the wild, remain important research topics to pursue. Biologging has the potential to address many of these lingering questions since accelerometers, along with the plethora of other sensors currently available, can inform on much more than buoyancy alone.

## Supplementary Material

10.1242/jexbio.252246_sup1 Supplementary information

## References

[JEB252246C1] Adachi, T., Maresh, J. L., Robinson, P. W., Peterson, S. H., Costa, D. P., Naito, Y., Watanabe, Y. Y. and Takahashi, A. (2014). The foraging benefits of being fat in a highly migratory marine mammal. *Proc. R. Soc. B* 281, 20142120. 10.1098/rspb.2014.2120 PMC4241001 25377461

[JEB252246C2] Alexander, R. M. (1990). Size, speed and buoyancy adaptations in aquatic animals. *Am. Zool.* 30, 189-196. 10.1093/icb/30.1.189

[JEB252246C3] Alexander, R. M. (1965). The lift produced by the heterocercal tails of selachii. *J. Exp. Biol.* 43, 131-138. 10.1242/jeb.43.1.131

[JEB252246C4] Andrzejaczek, S., Gleiss, A. C., Lear, K. O., Pattiaratchi, C. B., Chapple, T. K. and Meekan, M. G. (2019). Biologging tags reveal links between fine-scale horizontal and vertical movement behaviors in tiger sharks (*Galeocerdo cuvier*). *Front. Mar. Sci.* 6, 229. 10.3389/fmars.2019.00229

[JEB252246C5] Andrzejaczek, S., Gleiss, A. C., Lear, K. O., Pattiaratchi, C., Chapple, T. K. and Meekan, M. G. (2020). Depth-dependent dive kinematics suggest cost-efficient foraging strategies by tiger sharks. *R. Soc. Open Sci.* 7, 200789. 10.1098/rsos.200789 PMC7481696 32968529

[JEB252246C6] Biuw, M., McConnell, B., Bradshaw, C. J. A., Burton, H. and Fedak, M. (2003). Blubber and buoyancy: monitoring the body condition of free-ranging seals using simple dive characteristics. *J. Exp. Biol.* 206, 3405-3423. 10.1242/jeb.00583 12939372

[JEB252246C7] Bolker, B. M., Brooks, M. E., Clark, C. J., Geange, S. W., Poulsen, J. R., Stevens, M. H. H. and White, J.-S. S. (2009). Generalized linear mixed models: a practical guide for ecology and evolution. *Trends Ecol. Evol.* 24, 127-135. 10.1016/j.tree.2008.10.008 19185386

[JEB252246C8] Corner, E., Denton, E. J. and Forster, G. (1969). On the buoyancy of some deep-sea sharks. *Proc. R. Soc. Lond. B Biol. Sci.* 171, 415-429. 10.1098/rspb.1969.0003

[JEB252246C9] Denny, M. W. (1990). Terrestrial versus aquatic biology: the medium and its message. *Am. Zool.* 30, 111-121. 10.1093/icb/30.1.111

[JEB252246C10] Edwards, J. E., Hiltz, E., Broell, F., Bushnell, P. G., Campana, S. E., Christiansen, J. S., Devine, B. M., Gallant, J. J., Hedges, K. J., MacNeil, M. A. et al. (2019). Advancing research for the management of long-lived species: a case study on the Greenland shark. *Front. Mar. Sci.* 6, 87. 10.3389/fmars.2019.00087

[JEB252246C11] Edwards, J. E., Hedges, K. J. and Hussey, N. E. (2022). Seasonal residency, activity space, and use of deep-water channels by Greenland sharks (*Somniosus microcephalus*) in an Arctic fjord system. *Can. J. Fish. Aquat. Sci.* 79, 314-330. 10.1139/cjfas-2021-0009

[JEB252246C12] Elliott, K. H., Ricklefs, R. E., Gaston, A. J., Hatch, S. A., Speakman, J. R. and Davoren, G. K. (2013). High flight costs, but low dive costs, in auks support the biomechanical hypothesis for flightlessness in penguins. *Proc. Natl Acad. Sci. USA* 110, 9380-9384. 10.1073/pnas.1304838110 PMC3677478 23690614

[JEB252246C13] Ferry, L. A. and Lauder, G. V. (1996). Heterocercal tail function in leopard sharks: a three-dimensional kinematic analysis of two models. *J. Exp. Biol.* 199, 2253-2268. 10.1242/jeb.199.10.2253 9320170

[JEB252246C14] Fisk, A. T., Tittlemier, S. A., Pranschke, J. L. and Norstrom, R. J. (2002). Using anthropogenic contaminants and stable isotopes to assess the feeding ecology of Greenland sharks. *Ecology* 83, 2162-2172. 10.1890/0012-9658(2002)083[2162:UACASI]2.0.CO;2

[JEB252246C15] Furey, N. B., Hinch, S. G., Mesa, M. G. and Beauchamp, D. A. (2016). Piscivorous fish exhibit temperature-influenced binge feeding during an annual prey pulse. *J. Anim. Ecol.* 85, 1307-1317. 10.1111/1365-2656.12565 27457279

[JEB252246C16] Gallagher, A. J., Wagner, D. N., Irschick, D. J. and Hammerschlag, N. (2014). Body condition predicts energy stores in apex predatory sharks. *Conserv. Physiol.* 2, cou022. 10.1093/conphys/cou022 PMC4732496 27293643

[JEB252246C17] Gleiss, A. C., Norman, B. and Wilson, R. P. (2011). Moved by that sinking feeling: Variable diving geometry underlies movement strategies in whale sharks. *Funct. Ecol.* 25, 595-607. 10.1111/j.1365-2435.2010.01801.x

[JEB252246C18] Gleiss, A. C., Potvin, J., Keleher, J. J., Whitty, J. M., Morgan, D. L. and Goldbogen, J. A. (2015). Mechanical challenges to freshwater residency in sharks and rays. *J. Exp. Biol.* 218, 1099-1110. 10.1242/jeb.114868 25573824

[JEB252246C19] Gleiss, A. C., Potvin, J. and Goldbogen, J. A. (2017). Physical trade-offs shape the evolution of buoyancy control in sharks. *Proc. R. Soc. B* 284, 20171345. 10.1098/rspb.2017.1345 PMC5698638 29118132

[JEB252246C20] Gleiss, A. C., Schallert, R. J., Dale, J. J., Wilson, S. G. and Block, B. A. (2019). Direct measurement of swimming and diving kinematics of giant Atlantic bluefin tuna (*Thunnus thynnus*). *R. Soc. Open Sci.* 6, 190203. 10.1098/rsos.190203 PMC6549966 31218059

[JEB252246C21] Goulet, P., Guinet, C., Swift, R., Madsen, P. T. and Johnson, M. (2019). A miniature biomimetic sonar and movement tag to study the biotic environment and predator-prey interactions in aquatic animals. *Deep Sea Res. I: Oceanogr. Res. Pap.* 148, 1-11. 10.1016/j.dsr.2019.04.007

[JEB252246C22] Grant, K. R., Campbell, T. W., Silver, T. I. and Olea-Popelka, F. J. (2013). Validation of an ultrasound-guided technique to establish a liver-to-coelom ratio and a comparative analysis of the ratios among acclimated and recently wild-caught southern stingrays, *Dasyatis Americana*. *Zoo Biol.* 32, 104-111. 10.1002/zoo.21014 22588699

[JEB252246C23] Hussey, N. E., Cocks, D. T., Dudley, S. F. J., McCarthy, I. D. and Wintner, S. P. (2009). The condition conundrum: application of multiple condition indices to the dusky shark *Carcharhinus obscurus*. *Mar. Ecol. Prog. Ser.* 380, 199-212. 10.3354/meps07918

[JEB252246C24] Iosilevskii, G. and Papastamatiou, Y. P. (2016). Relations between morphology, buoyancy and energetics of requiem sharks. *R. Soc. Open Sci.* 3, 160406. 10.1098/rsos.160406 PMC5098981 27853556

[JEB252246C25] Iosilevskii, G., Papastamatiou, Y. P., Meyer, C. G. and Holland, K. N. (2012). Energetics of the yo-yo dives of predatory sharks. *J. Theor. Biol.* 294, 172-181. 10.1016/j.jtbi.2011.11.008 22108240

[JEB252246C26] Irschick, D. J. and Hammerschlag, N. (2014). A new metric for measuring condition in large predatory sharks. *J. Fish Biol.* 85, 917-926. 10.1111/jfb.12484 25130454

[JEB252246C27] Johnson, M. P. and Tyack, P. L. (2003). A digital acoustic recording tag for measuring the response of wild marine mammals to sound. *IEEE J. Ocean. Eng.* 28, 3-12. 10.1109/JOE.2002.808212

[JEB252246C28] Kawatsu, S., Sato, K., Watanabe, Y., Hyodo, S., Breves, J. P., Fox, B. K., Grau, E. G. and Miyazaki, N. (2009). A new method to calibrate attachment angles of data loggers in swimming sharks. *EURASIP J. Adv. Signal Process.* 2010, 1-6. 10.1155/2010/732586

[JEB252246C29] Leclerc, L.-M. E., Lydersen, C., Haug, T., Bachmann, L., Fisk, A. T. and Kovacs, K. M. (2012). A missing piece in the Arctic food web puzzle? Stomach contents of Greenland sharks sampled in Svalbard, Norway. *Polar Biol.* 35, 1197-1208. 10.1007/s00300-012-1166-7

[JEB252246C30] Lindmark, M., Anderson, S. C., Gogina, M. and Casini, M. (2023). Evaluating drivers of spatiotemporal variability in individual condition of a bottom-associated marine fish, Atlantic cod (*Gadus morhua*). *ICES J. Mar. Sci.* 80, 1539-1550. 10.1093/icesjms/fsad084

[JEB252246C31] MacNeil, M. A., McMeans, B. C., Hussey, N. E., Vecsei, P., Svavarsson, J., Kovacs, K. M., Lydersen, C., Treble, M. A., Skomal, G. B., Ramsey, M. et al. (2012). Biology of the Greenland shark *Somniosus microcephalus*. *J. Fish Biol.* 80, 991-1018. 10.1111/j.1095-8649.2012.03257.x 22497371

[JEB252246C32] McMeans, B. C., Arts, M. T., Lydersen, C., Kovacs, K. M., Hop, H., Falk-Petersen, S. and Fisk, A. T. (2013). The role of Greenland sharks (*Somniosus microcephalus*) in an Arctic ecosystem: assessed via stable isotopes and fatty acids. *Mar. Biol.* 160, 1223-1238. 10.1007/s00227-013-2174-z

[JEB252246C33] Miller, P. J. O., Biuw, M., Watanabe, Y. Y., Thompson, D. and Fedak, M. A. (2012). Sink fast and swim harder! Round-trip cost-of-transport for buoyant divers. *J. Exp. Biol.* 215, 3622-3630. 10.1242/jeb.070128 23014571

[JEB252246C34] Molde, K., Ciesielski, T. M., Fisk, A. T., Lydersen, C., Kovacs, K. M., Sørmo, E. G. and Jenssen, B. M. (2013). Associations between vitamins A and E and legacy POP levels in highly contaminated Greenland sharks (*Somniosus microcephalus*). *Sci. Total Environ.* 442, 445-454. 10.1016/j.scitotenv.2012.10.012 23183125

[JEB252246C35] Musick, J. A. and Cotton, C. F. (2015). Bathymetric limits of chondrichthyans in the deep sea: a re-evaluation. *Deep Sea Res. II: Top. Stud. Oceanogr. Biol. Deep-Water Chondrichthyans* 115, 73-80. 10.1016/j.dsr2.2014.10.010

[JEB252246C36] Nakamura, I., Meyer, C. G. and Sato, K. (2015). Unexpected positive buoyancy in deep sea sharks, *Hexanchus griseus*, and a *Echinorhinus cookei*. *PLoS ONE* 10, e0127667. 10.1371/journal.pone.0127667 PMC4489517 26061525

[JEB252246C37] Nakamura, I., Watanabe, Y. Y., Papastamatiou, Y. P., Sato, K. and Meyer, C. G. (2011). Yo-yo vertical movements suggest a foraging strategy for tiger sharks *Galeocerdo cuvier*. *Mar. Ecol. Prog. Ser.* 424, 237-246. 10.3354/meps08980

[JEB252246C38] Nathan, R., Getz, W. M., Revilla, E., Holyoak, M., Kadmon, R., Saltz, D. and Smouse, P. E. (2008). A movement ecology paradigm for unifying organismal movement research. *Proc. Natl Acad. Sci. USA* 105, 19052-19059. 10.1073/pnas.0800375105 PMC2614714 19060196

[JEB252246C39] Nielsen, J., Christiansen, J. S., Grønkjær, P., Bushnell, P., Steffensen, J. F., Kiilerich, H. O., Præbel, K. and Hedeholm, R. (2019). Greenland shark (*Somniosus microcephalus*) stomach contents and stable isotope values reveal an ontogenetic dietary shift. *Front. Mar. Sci.* 6, 125. 10.3389/fmars.2019.00125

[JEB252246C40] Nielsen, J., Hedeholm, R. B., Lynghammar, A., McClusky, L. M., Berland, B., Steffensen, J. F. and Christiansen, J. S. (2020). Assessing the reproductive biology of the Greenland shark (*Somniosus microcephalus*). *PLoS ONE* 15, e0238986. 10.1371/journal.pone.0238986 PMC7540863 33027263

[JEB252246C41] Nowacek, D. P., Johnson, M. P., Tyack, P. L., Shorter, K. A., McLellan, W. A. and Pabst, D. A. (2001). Buoyant balaenids: The ups and downs of buoyancy in right whales. *Proc. R. Soc. B* 268, 1811-1816. 10.1098/rspb.2001.1730 PMC1088813 11522200

[JEB252246C42] Pelster, B. (2009). Buoyancy control in aquatic vertebrates. In *Cardio-Respiratory Control in Vertebrates: Comparative and Evolutionary Aspects* (ed. M. L. Glass and S. C. Wood), pp. 65-98. Springer. 10.1007/978-3-540-93985-6_4

[JEB252246C43] Pinte, N., Godefroid, M., Abbas, O., Baeten, V. and Mallefet, J. (2019). Deep-sea sharks: relation between the liver's buoyancy and red aerobic muscle volumes, a new approach. *Comp. Biochem. Physiol. A Mol. Integr. Physiol.* 236, 110520. 10.1016/j.cbpa.2019.06.020 31278989

[JEB252246C44] Priede, I. G., Burgass, R. W., Mandalakis, M., Spyros, A., Gikas, P., Burns, F. and Drewery, J. (2020). Near-equal compressibility of liver oil and seawater minimises buoyancy changes in deep-sea sharks and chimaeras. *J. Exp. Biol.* 223, jeb222943. 10.1242/jeb.222943 32291325

[JEB252246C45] Sakamoto, K. Q., Sato, K., Ishizuka, M., Watanuki, Y., Takahashi, A., Daunt, F. and Wanless, S. (2009). Can ethograms be automatically generated using body acceleration data from free-ranging birds? *PLoS ONE* 4, e5379. 10.1371/journal.pone.0005379 PMC2671159 19404389

[JEB252246C46] Saraiva, B. M., Macena, B. C. L., Solleliet-Ferreira, S., Afonso, P. and Fontes, J. (2023). First insights into the shortfin mako shark (*Isurus oxyrinchus*) fine-scale swimming behaviour. *R. Soc. Open Sci.* 10, 230012. 10.1098/rsos.230012 PMC10154923 37153366

[JEB252246C47] Sato, K., Naito, Y., Kato, A., Niizuma, Y., Watanuki, Y., Charrassin, J. B., Bost, C.-A., Handrich, Y. and Le Maho, Y. (2002). Buoyancy and maximal diving depth in penguins: Do they control inhaling air volume? *J. Exp. Biol.* 205, 1189-1197. 10.1242/jeb.205.9.1189 11948196

[JEB252246C48] Sato, K., Mitani, Y., Cameron, M. F., Siniff, D. B. and Naito, Y. (2003). Factors affecting stroking patterns and body angle in diving Weddell seals under natural conditions. *J. Exp. Biol.* 206, 1461-1470. 10.1242/jeb.00265 12654885

[JEB252246C49] Sato, K., Aoki, K., Watanabe, Y. Y. and Miller, P. J. O. (2013). Neutral buoyancy is optimal to minimize the cost of transport in horizontally swimming seals. *Sci. Rep.* 3, 2205. 10.1038/srep02205 PMC3712316 23857645

[JEB252246C50] Schilling, J. W., Hussey, N. E., Hedges, K. J. and Barkley, A. N. (2025). Non-invasive ultrasound measurements for determination of hepato-somatic index in Greenland halibut (*Reinhardtius hippoglossoides*). *J. Fish Biol.* 107, 1313-1322. 10.1111/jfb.70089 PMC12536057 40574286

[JEB252246C51] Shepard, E. L. C., Wilson, R. P., Quintana, F., Laich, A. G., Liebsch, N., Albareda, D. A., Halsey, L. G., Gleiss, A., Morgan, D. T., Myers, A. E. et al. (2008). Identification of animal movement patterns using tri-axial accelerometry. *Endanger. Species Res.* 10, 47-60. 10.3354/esr00084

[JEB252246C52] Shepard, E., Wilson, R., Gómez Laich, A. and Quintana, F. (2010). Buoyed up and slowed down: Speed limits for diving birds in shallow water. *Aquat. Biol.* 8, 259-267. 10.3354/ab00232

[JEB252246C53] Ste-Marie, E., Watanabe, Y. Y., Semmens, J. M., Marcoux, M. and Hussey, N. E. (2020). A first look at the metabolic rate of Greenland sharks (*Somniosus microcephalus*) in the Canadian Arctic. *Sci. Rep.* 10, Article 1. 10.1038/s41598-020-76371-0 PMC7653932 33168918

[JEB252246C54] Ste-Marie, E., Watanabe, Y. Y., Semmens, J. M., Marcoux, M. and Hussey, N. E. (2022). Life in the slow lane: Field metabolic rate and prey consumption rate of the Greenland shark (*Somniosus microcephalus*) modelled using archival biologgers. *J. Exp. Biol.* 225, jeb242994. 10.1242/jeb.242994 35258589

[JEB252246C55] Treberg, J. R. and Speers-Roesch, B. (2016). Does the physiology of chondrichthyan fishes constrain their distribution in the deep sea? *J. Exp. Biol.* 219, 615-625. 10.1242/jeb.128108 26936637

[JEB252246C56] Watanabe, Y., Baranov, E. A., Sato, K., Naito, Y. and Miyazaki, N. (2004). Foraging tactics of Baikal seals differ between day and night. *Mar. Ecol. Prog. Ser.* 279, 283-289. 10.3354/meps279283

[JEB252246C57] Watanabe, Y. Y., Baranov, E. A., Sato, K., Naito, Y. and Miyazaki, N. (2006). Body density affects stroke patterns in Baikal seals. *J. Exp. Biol.* 209, 3269-3280. 10.1242/jeb.02402 16916962

[JEB252246C58] Watanabe, Y. Y., Lydersen, C., Fisk, A. T. and Kovacs, K. M. (2012). The slowest fish: swim speed and tail-beat frequency of Greenland sharks. *J. Exp. Mar. Biol. Ecol.* 426-427, 5-11. 10.1016/j.jembe.2012.04.021

[JEB252246C59] Watanabe, Y. Y. and Papastamatiou, Y. P. (2023). Biologging and biotelemetry: tools for understanding the lives and environments of marine animals. *Ann. Rev. Anim. Biosci.* 11, 247-267. 10.1146/annurev-animal-050322-073657 36790885

[JEB252246C60] Watanabe, Y. Y., Payne, N. L., Semmens, J. M., Fox, A. and Huveneers, C. (2019). Swimming strategies and energetics of endothermic white sharks during foraging. *J. Exp. Biol.* 222, jeb185603. 10.1242/jeb.185603 30777873

[JEB252246C61] Watanabe, Y. Y., Stamp, T., Barkley, A. N., Hedges, K. J. and Hussey, N. E. (2026). Post-release behaviour, physiological stress, and survival rates of longline-caught Greenland sharks. *Conserv. Physiol.* 14, coag031. 10.1093/conphys/coag031 PMC13201077 42199783

[JEB252246C62] Weihs, D. (1973). Mechanically efficient swimming techniques for fish with negative buoyancy. *J. Mar. Res.* 31, 194-209.

[JEB252246C63] Whitney, N. M., White, C. F., Gleiss, A. C., Schwieterman, G. D., Anderson, P., Hueter, R. E. and Skomal, G. B. (2016). A novel method for determining post-release mortality, behavior, and recovery period using acceleration data loggers. *Fish. Res.* 183, 210-221. 10.1016/j.fishres.2016.06.003

[JEB252246C64] Williams, T. M., Davis, R. W., Fuiman, L. A., Francis, J., Le Boeuf, B. J., Horning, M., Calambokidis, J. and Croll, D. A. (2000). Sink or swim: strategies for cost-efficient diving by marine mammals. *Science* 288, 133-136. 10.1126/science.288.5463.133 10753116

[JEB252246C65] Wilson, R., Shepard, E., Gómez Laich, A., Frere, E. and Quintana, F. (2010). Pedalling downhill and freewheeling up; a penguin perspective on foraging. *Aquat. Biol.* 8, 193-202. 10.3354/ab00230

[JEB252246C66] Withers, P. C., Morrison, G., Hefter, G. T. and Pang, T.-S. (1994). Role of urea and methylamines in buoyancy of elasmobranchs. *J. Exp. Biol.* 188, 175-189. 10.1242/jeb.188.1.175 9317582

