# Supplementary material for: Greenland sharks conform to expectations of neutral buoyancy but exhibit inter-individual variation
Source: J Exp Biol. 2026 Sep 25;229(18):jeb252246. doi: 10.1242/jeb.252246 (PMC13680965; doi:10.1242/jeb.252246)
Supplement: Supplementary file 1 [file jexbio-229-252246-s1.pdf]

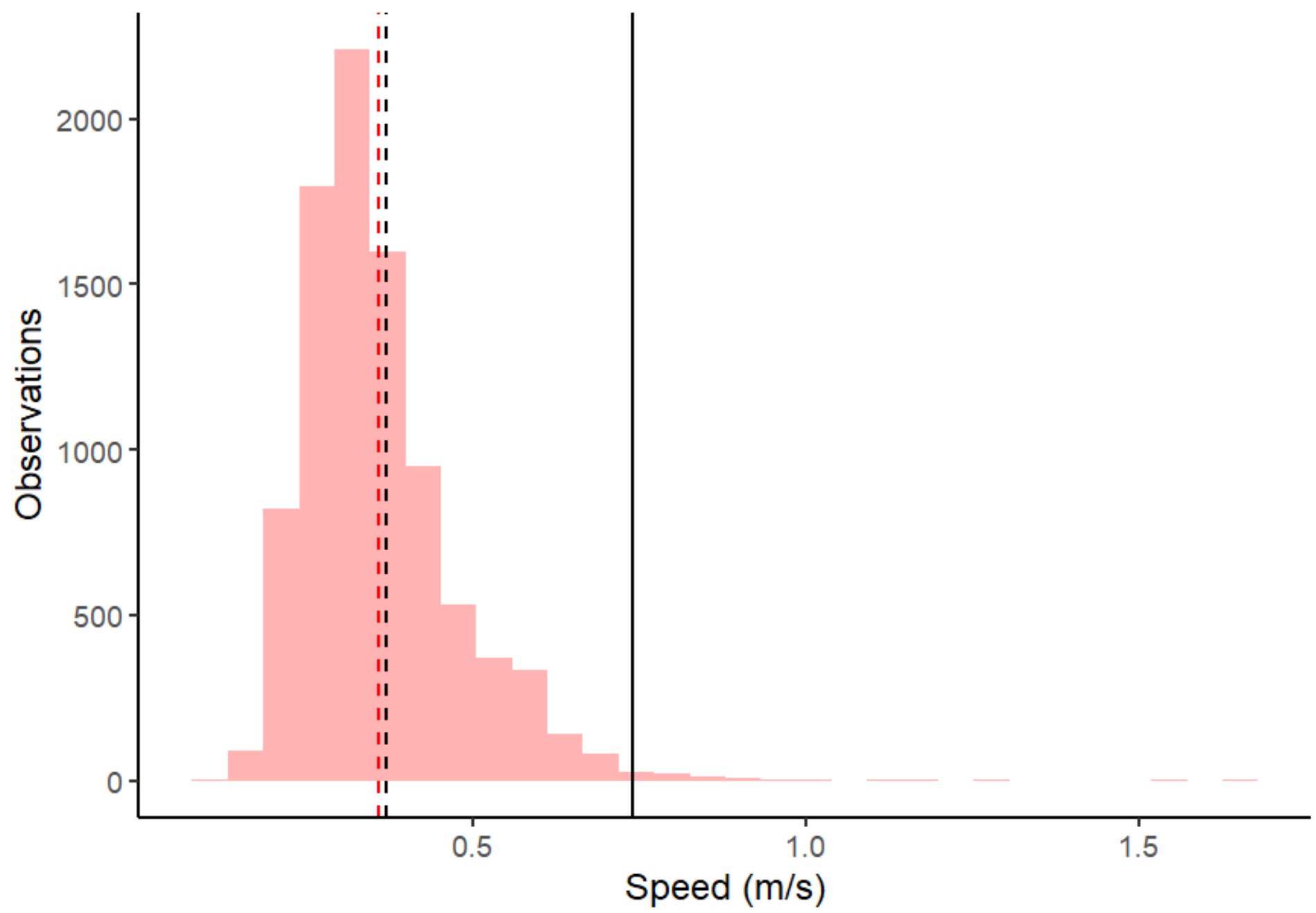

Fig. S1. Histogram of derived speed estimates for all dive segments at a pitch of 5 degrees or more. The red dashed line represents the average speed estimated for Greenland sharks in the current dataset. The dashed and solid black lines represent the average and maximum speed measured by loggers in a previous study on Greenland sharks (Watanabe et al., 2012).

Journal of Experimental Biology • Supplementary information

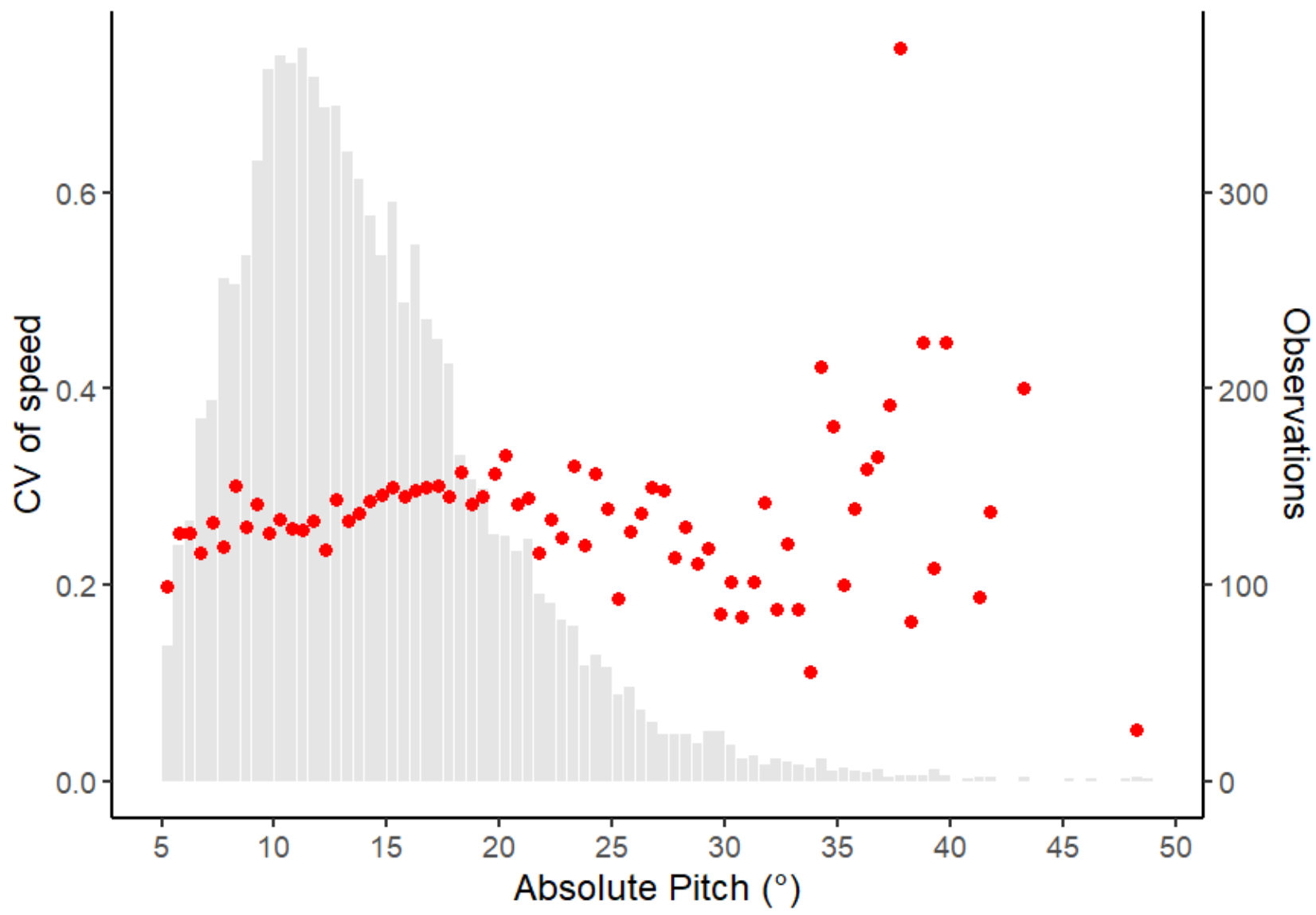

Fig. S2. Coefficient of variation (i.e. sd/mean; red points) for speed estimates sorted into 0.5 degree absolute pitch bins. A histogram of the number of observations within each pitch bin appears in the background. Note: CV does not increase at low pitch angles, as might be expected if speed estimates were unreliable at low pitch angles.

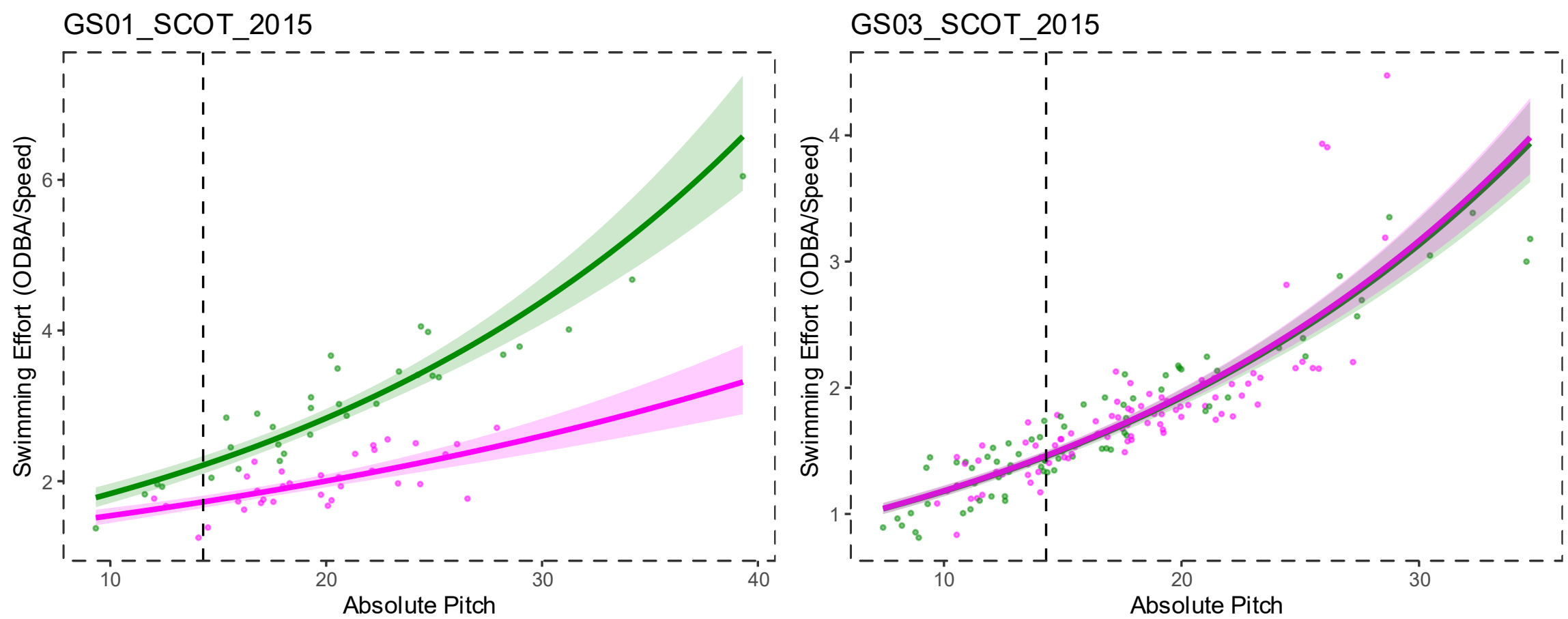

16

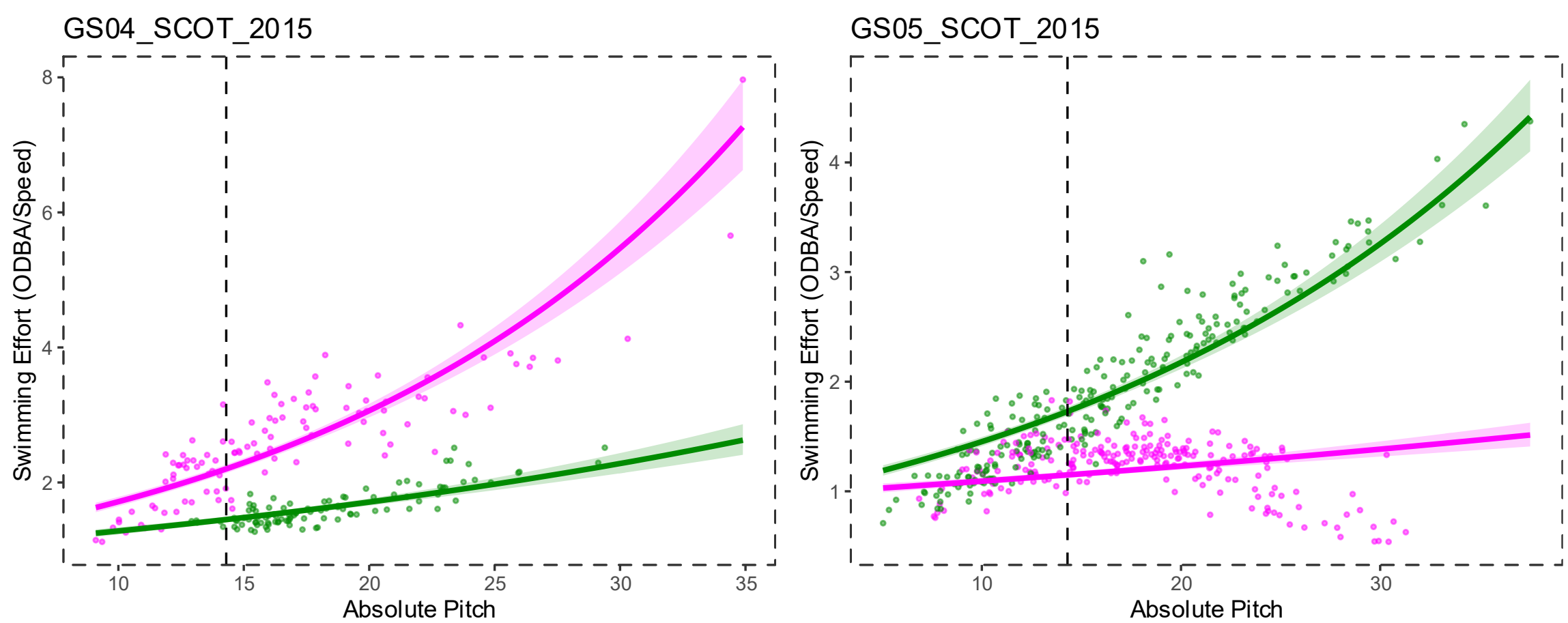

17

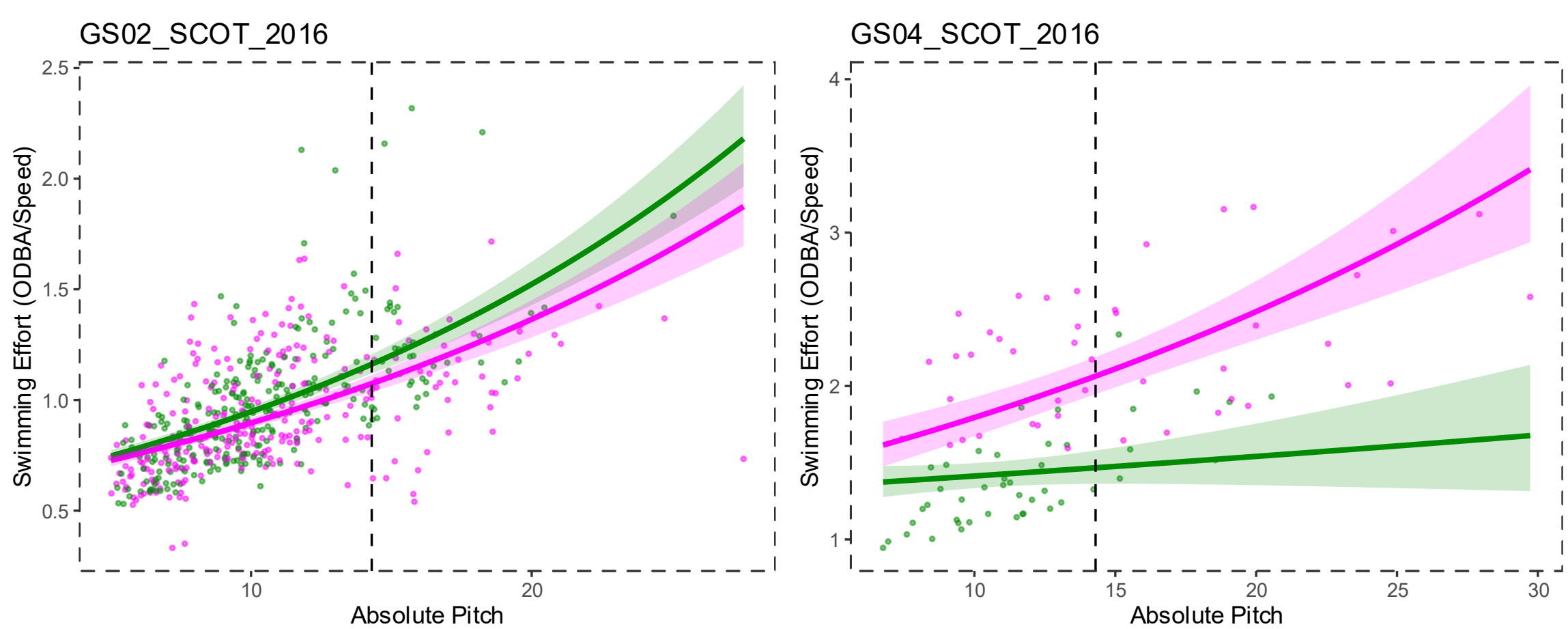

Journal of Experimental Biology • Supplementary information

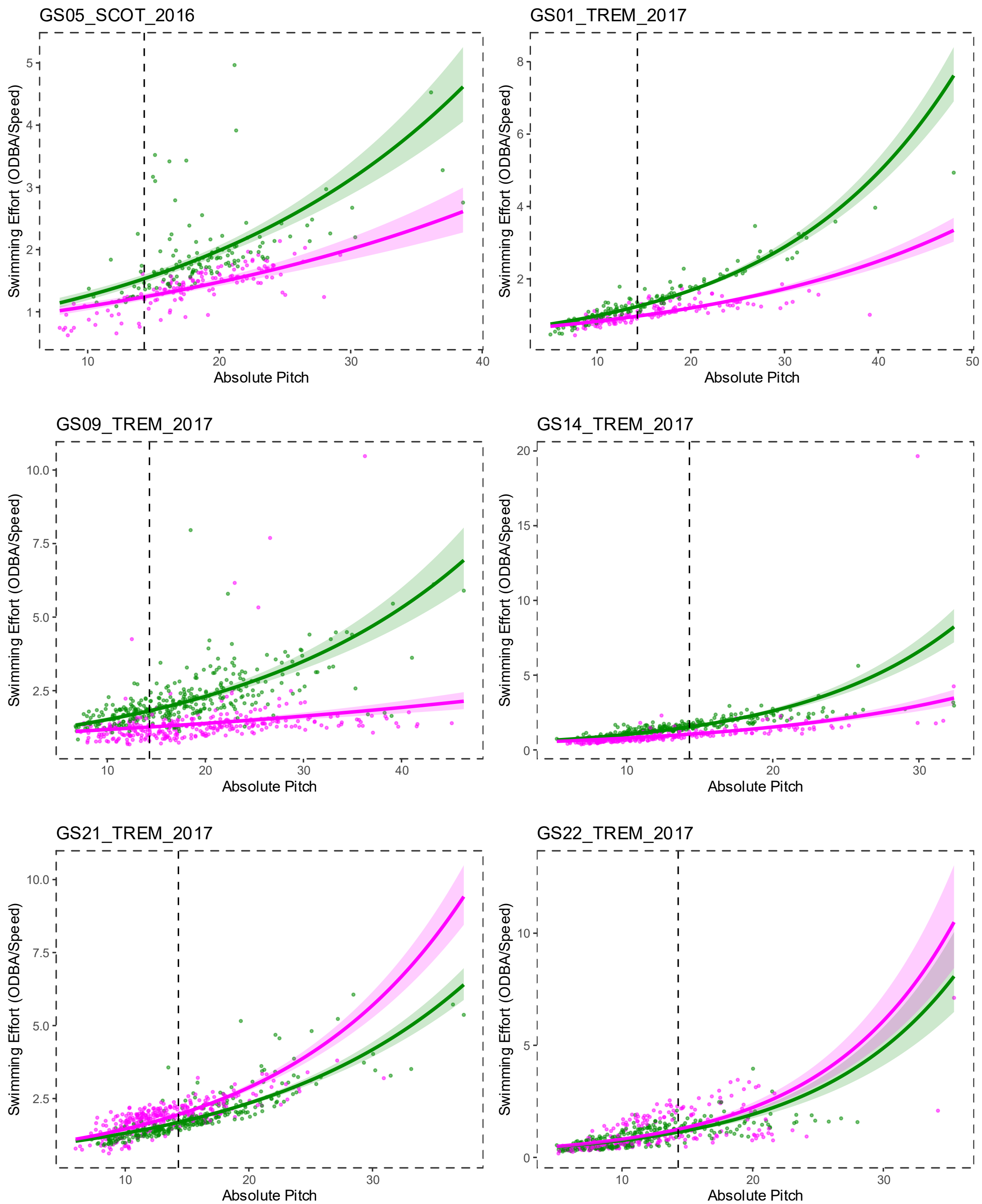

Journal of Experimental Biology • Supplementary information

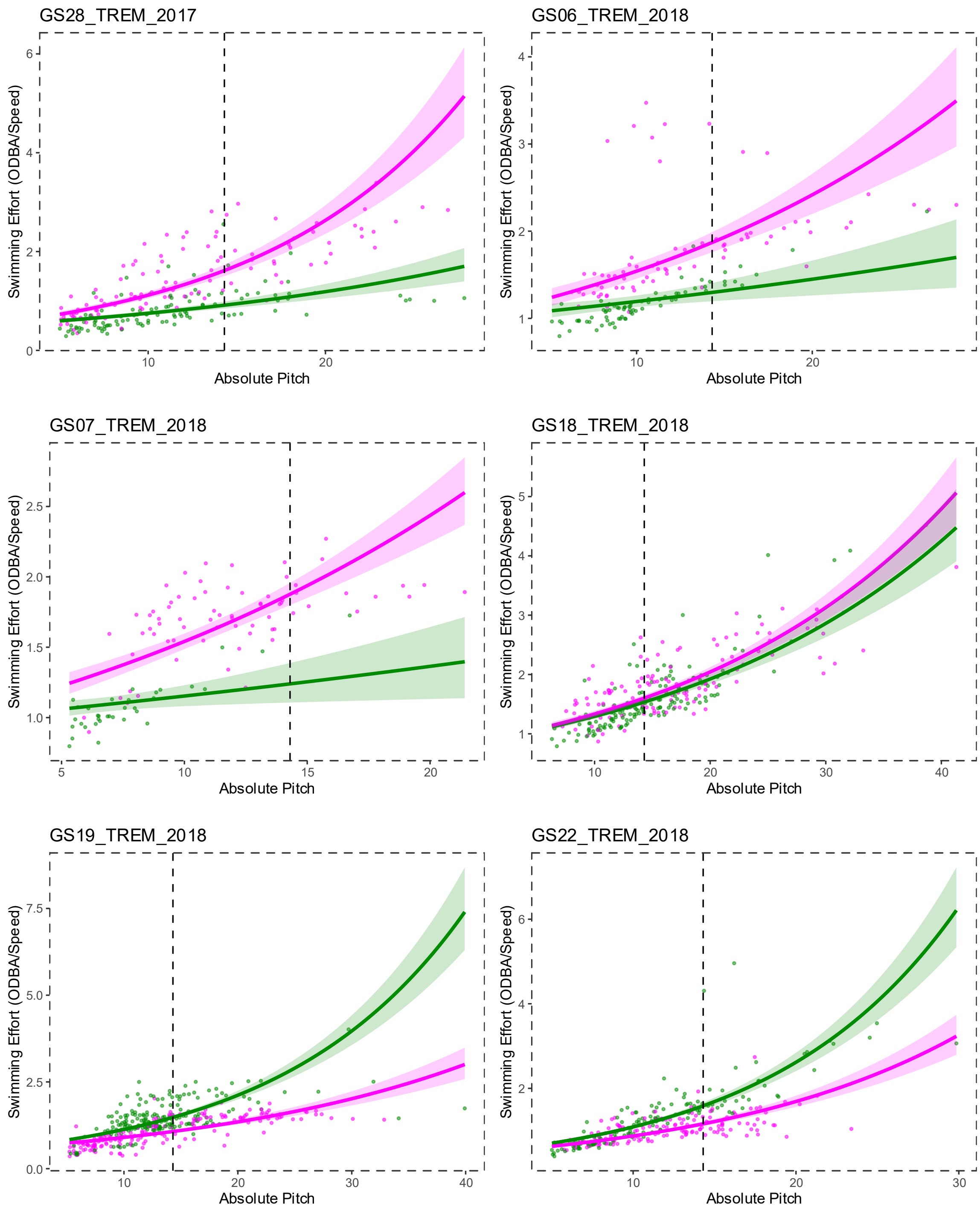
GS28_TREM_2017
GS06_TREM_2018
GS07_TREM_2018
GS18_TREM_2018
GS19_TREM_2018
GS22_TREM_2018
Swimming Effort (ODBA/Speed)
Absolute Pitch

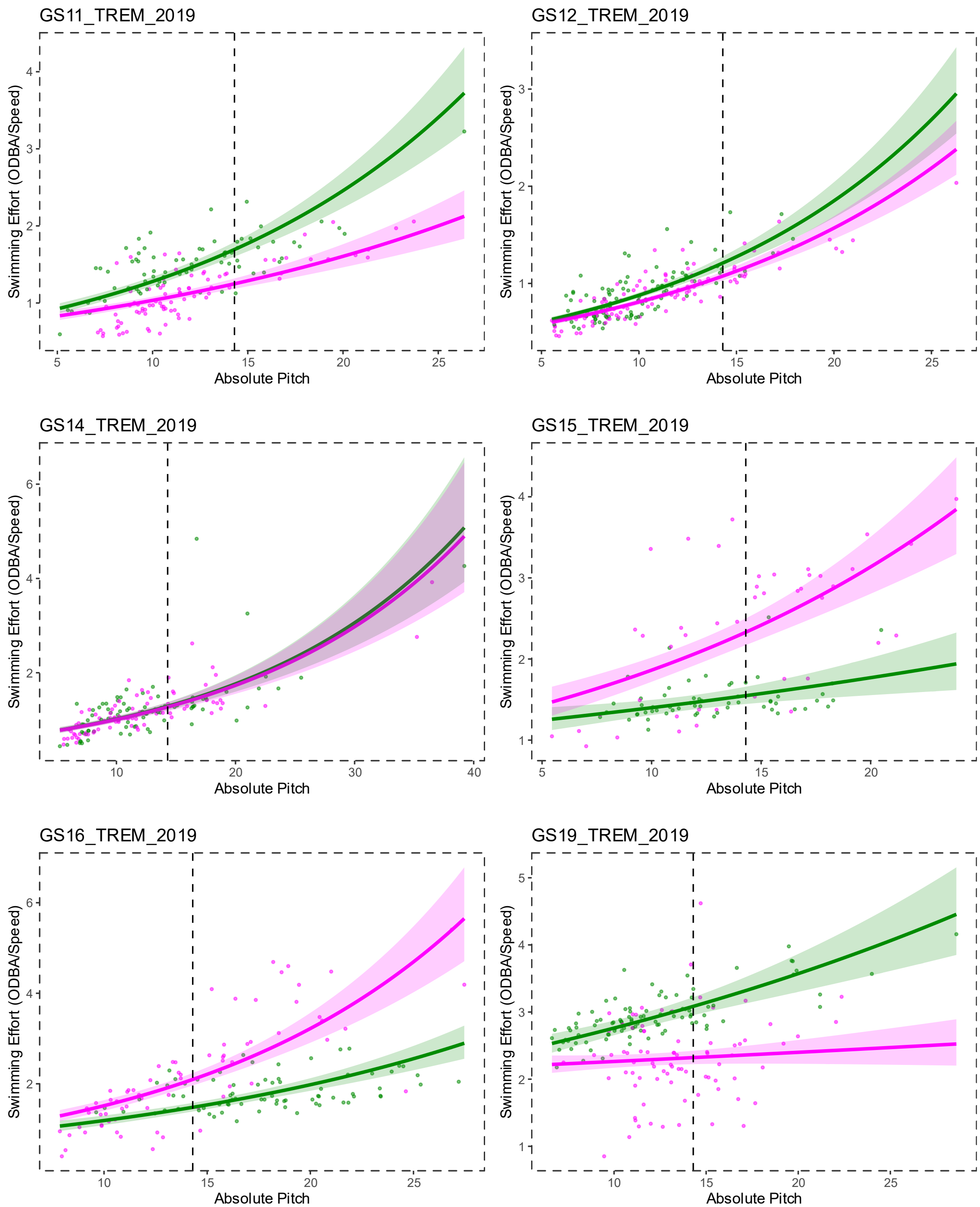

Journal of Experimental Biology • Supplementary information

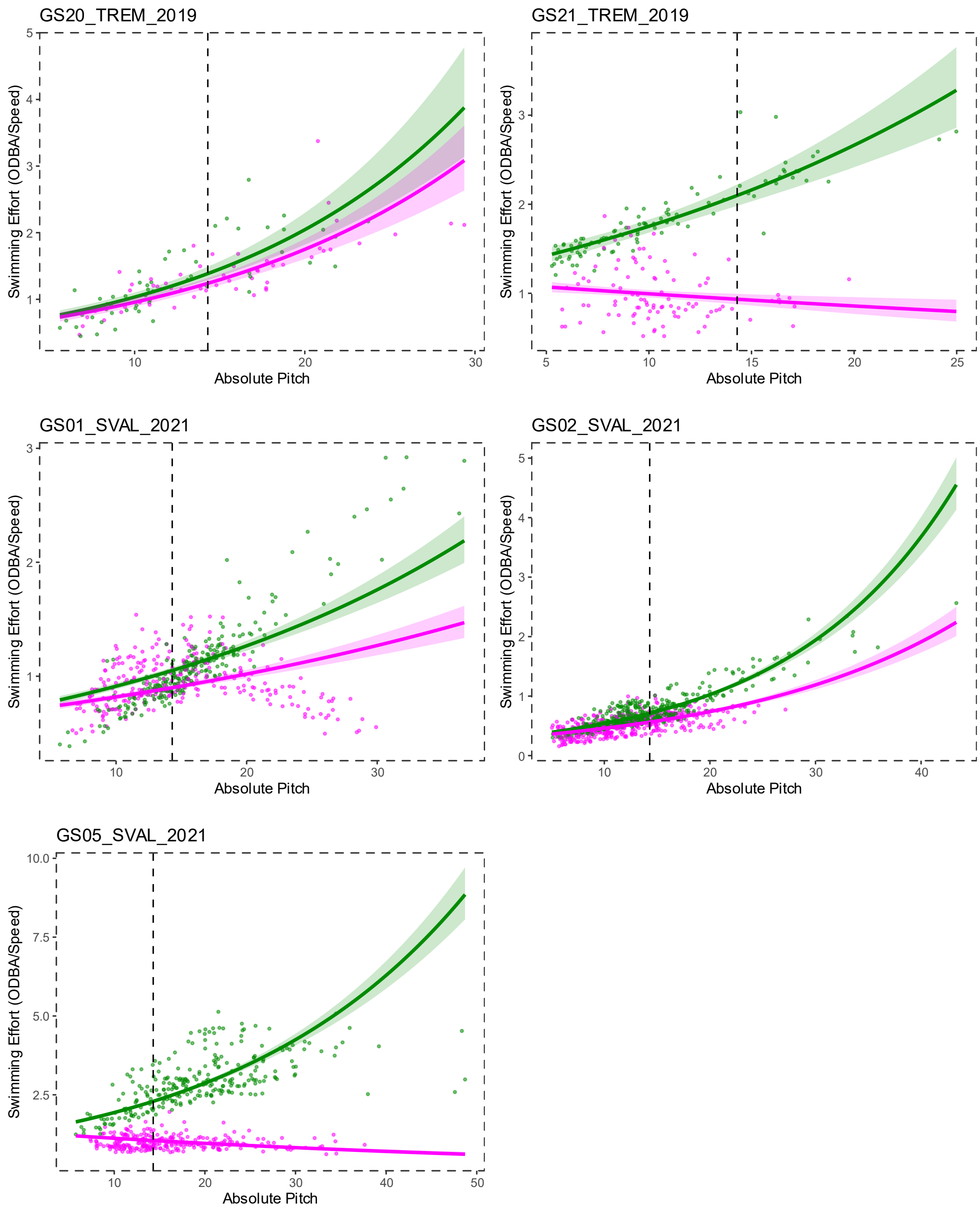

Fig. S3. Plots depicting swimming effort during individual dive segments against absolute pitch for all greenland sharks included in analyses. Colour signifies dive direction (ascending [green] or descending [magenta]) and lines represent the output of log link generalized linear models. The dashed vertical line indicates an absolute pitch of 14.3° (i.e. value used to estimate relative buoyancy ratios for comparisons across individuals based on the average across all sharks).

Journal of Experimental Biology • Supplementary information
